# Testicular and Rumen Microbial Responses to Selenium-Enriched Malt in Heat-Exposed Simmental Bulls

**DOI:** 10.3390/ani16213485

**Published:** 2026-11-04

**Authors:** Na Li, Fan Li, Mingqi Zhang, Fanfan Zhang, Chengcheng Wu, Lizhen Hu, Jun Xu

**Affiliations:** 1 Institute of Animal Husbandry and Veterinary Medicine, Jiangxi Academy of Agricultural Sciences, Nanchang 330200, China; 2 Jiangxi Provincial Key Laboratory of Green and Healthy Breeding of Livestock and Poultry, Nanchang 330200, China

**Keywords:** selenium, heat stress, testis, reproductive endocrinology, rumen microbiome, metagenomics

## Abstract

This study evaluated testicular and rumen microbial responses to selenium-enriched malt in heat-exposed Simmental bulls. Eighteen bulls received a basal diet alone or supplemented to provide target supplemental Se levels of 0.3 or 0.5 mg/kg dietary DM. Metagenomics included six bulls per group, host assays four per group, and RNA sequencing three per group. Compared with controls, the high-dose group had higher serum antioxidant indices and reproductive hormone concentrations, lower inflammatory markers, higher Johnsen scores, and lower TUNEL positivity. Gene-level FDR control identified 23, 1, and 4 differentially expressed genes in the low-dose versus control, high-dose versus control, and high-dose versus low-dose contrasts, respectively. Provider-reported GSEA showed negative normalized enrichment scores for steroid biosynthesis in the control-versus-low-dose and control-versus-high-dose contrasts, indicating enrichment toward the supplemented side under the provider’s contrast convention. qPCR showed lower CYP11A1, CYP17A1, SCARB1, and LDLR expression at the low dose, whereas Bax, Bcl-2, and CYP17A1 protein abundance did not differ. Rumen microbial diversity and community composition differed among groups, and two post hoc selected cofactor-related pathways had lower abundance at the low dose. None of the 160 microbiome–testis or 720 microbiome–serum associations survived FDR correction. Supplementation was associated with favorable systemic and testicular histological responses and dose-specific molecular and microbial changes under heat exposure.

## Simple Summary

Heat exposure can compromise testicular health in cattle, highlighting the need for nutritional strategies that support reproductive tissues. We examined responses to selenium-enriched malt supplementation in 18 Simmental bulls exposed to environmental heat. Bulls receiving the higher supplementation level had higher serum antioxidant indices and reproductive hormone concentrations, lower inflammatory markers, better testicular histological scores, and fewer cells with detectable DNA fragmentation. Supplementation was also accompanied by dose-specific changes in testicular gene expression and rumen microbial diversity and functional profiles. These findings characterize favorable systemic and testicular responses associated with selenium-enriched malt supplementation in heat-exposed bulls and provide a basis for further evaluation of its use in cattle nutrition.

## 1. Introduction

Heat exposure is a challenge to cattle production because it can disrupt physiological regulation and impair reproductive tissues. Male reproduction depends on effective testicular thermoregulation, and excessive heat can damage germ cells and disturb spermatogenesis [1,2,3]. Experimental testicular hyperthermia also alters local testosterone concentrations and steroidogenic gene expression in bulls [4]. Systemic oxidative and inflammatory responses may accompany these changes [2,5]. Nutritional approaches that support physiological function during environmental heat exposure are therefore relevant to cattle management.

Selenium (Se) supports antioxidant defense and reproductive physiology through selenoproteins involved in cellular redox regulation [6,7,8]. Studies of heat-exposed cattle have reported responses to selenium-containing nutritional interventions, including selenium yeast in fattening bulls and hydroxy-selenomethionine in dairy cows [9,10]. However, direct testicular responses to selenium-enriched malt under natural heat exposure remain incompletely characterized. Cholesterol transport and steroidogenesis provide relevant molecular targets, but transcript abundance alone cannot establish changes in steroid production.

The rumen microbiome may also respond to dietary selenium and environmental heat. Heat exposure has been associated with microbial compositional and functional differences in cattle [11,12]. Sodium selenite supplementation altered ruminal microbial populations and fermentation-related traits in Holstein bulls [13], while work in beef cattle has linked selenium-containing interventions with microbial and host antioxidant responses [14]. These observations motivate joint assessment of host and rumen traits. They do not establish that rumen microbial changes mediate the testicular response to selenium-enriched malt.

Gastrointestinal microbial communities have been associated with male reproductive physiology through metabolic, immune, and endocrine interactions [15]. Much of this evidence comes from humans or non-ruminant models, leaving its relevance to the bovine rumen uncertain. Given the involvement of selenium-dependent proteins in antioxidant defense, redox regulation, and male reproductive physiology, selenium-enriched malt was selected as a nutritional intervention for evaluation under environmental heat exposure. We therefore evaluated serum indices, testicular histology, molecular measurements, and rumen shotgun metagenomics in Simmental bulls receiving selenium-enriched malt during natural heat exposure. We hypothesized that supplementation would be associated with more favorable systemic and testicular responses, together with dose-specific changes in testicular gene expression and rumen microbial profiles. Individually matched microbiome–host correlations were treated as exploratory.

## 2. Materials and Methods

### 2.1. Animals, Experimental Design, and Selenium Supplementation

Eighteen healthy Simmental bulls (initial body weight, approximately 500 ± 50 kg) were allocated by randomly drawing animal identification numbers without replacement to three treatments (*n* = 6 per treatment): a heat-exposed control group (HS), a low-dose selenium-enriched malt group (HS + Se-L), and a high-dose selenium-enriched malt group (HS + Se-H). The experiment was conducted at a commercial cattle farm in Gao’an, Yichun, Jiangxi Province, China. Before the feeding trial, all bulls underwent a 14-day adaptation period under the same feeding and management conditions subsequently used during the experiment. Each bull was housed in a separate pen, fed twice daily at approximately 06:00 and 18:00, and provided with free access to fresh drinking water.

All bulls received the same roughage-based basal diet throughout the experiment (Table 1). The selenium-enriched malt supplement was supplied as a powder by Feiyue Biotechnology Co., Ltd. (Zhengzhou, China) and had a manufacturer-specified selenium concentration of 2000 mg/kg. For the HS + Se-L and HS + Se-H groups, the daily amounts of selenium-enriched malt powder were calculated based on an estimated dry matter intake of 10 kg per bull per day. The calculated amounts were intended to provide target supplemental selenium levels of 0.3 and 0.5 mg/kg dietary DM, respectively. Each daily dose was weighed separately for each bull, administered through the individual feeding trough, and confirmed to be completely consumed. Bulls in the HS group received no selenium-enriched malt.

**Table 1:** Ingredient composition and nutrient levels of the basal diet.

Ingredient	% of Diet	Nutrient	Level
Whole-plant corn silage	44.00	Dry matter, %	52.30
Corn	16.00	Combined net energy, MJ/kg	6.38
Brewer’s grains	12.00	Crude protein, %	15.75
Peanut vines	7.00	Crude fat, %	1.18
Soybean residue	6.00	Crude fiber, %	25.58
Wheat bran	5.00	Ash, %	8.24
Wheat straw	4.50	Nitrogen-free extract, %	49.23
Soybean meal	4.00	Neutral detergent fiber, %	52.24
Sodium bicarbonate	0.50	Acid detergent fiber, %	42.88
Premix	1.00	Calcium, %	0.52
Total	100.00	Phosphorus, %	0.33

Ingredient proportions are presented on an as-fed basis, and nutrient levels are expressed on a DM basis unless otherwise indicated. Net energy and nitrogen-free extract were calculated, whereas the other nutrient values were measured. All groups received the same basal diet with target supplemental Se levels of 0, 0.3, or 0.5 mg/kg DM for HS, HS + Se-L, and HS + Se-H, respectively.

The 56-day feeding trial was conducted during a period of naturally occurring summer heat from July to September 2024. Ambient temperature and relative humidity (RH) were recorded at 15-min intervals using an environmental data logger. The temperature–humidity index (THI) was calculated according to Hahn [16] as follows:

THI = 0.81 × *T* + (*RH*/100) × (*T* − 14.4) + 46.4

where *T* is the ambient temperature (°C) and *RH* is the relative humidity expressed as a percentage. Mean ambient temperature and *RH* were 29.21 ± 2.22 °C and 83.23 ± 7.38%, respectively, and mean THI was 82.25 ± 2.59 (range, 76.97–94.96). THI exceeded 74 at all recorded time points and exceeded 78 at 98.5% of the recorded time points, indicating sustained environmental heat exposure throughout the feeding trial.

Body weight was recorded at the beginning and end of the experiment. The general condition of the bulls was monitored during routine twice-daily feeding, and no bull was removed because of illness or other health-related conditions. All experimental procedures were conducted in accordance with institutional guidelines for animal care and use and were approved by the Experimental Animal Ethics Committee of the Institute of Animal Husbandry and Veterinary Medicine, Jiangxi Academy of Agricultural Sciences (Approval No. 2024-JXAAS-XM-09; approved in February 2024).

### 2.2. Sample Collection

At the end of the experimental period, four bulls per treatment were randomly selected for the host assays before the corresponding laboratory outcomes were available. The host-assay subset size was determined before laboratory analysis based on assay capacity and available resources and was not selected on the basis of observed outcomes. Before the morning feeding, approximately 10 mL of blood was collected from the jugular vein of each selected bull into an additive-free vacuum tube. Samples were allowed to clot and centrifuged at 3000 rpm for 10 min, and the resulting serum was stored at −80 °C until analysis.

At the end of the feeding trial and before morning feeding, rumen fluid was collected from all 18 bulls using an oral rumen-fluid sampler. Samples intended for metagenomic analysis were immediately frozen and stored at −80 °C. All bulls were subsequently slaughtered at a commercial abattoir following mechanical stunning and exsanguination. Testicular tissues were collected immediately after slaughter from the four selected bulls per treatment. Portions intended for RNA and protein analyses were snap-frozen in liquid nitrogen and stored at −80 °C, whereas portions intended for histological examination and TUNEL staining were fixed in 4% paraformaldehyde for 48 h and subsequently embedded in paraffin.

Serum assays, histological assessment, TUNEL staining, quantitative real-time PCR, and Western blotting were performed using the same four bulls per treatment. RNA sequencing was performed using three prespecified bulls from each of these subsets, selected before the RNA-seq or other host-assay outcomes were examined. Microbiome–host association analyses included the 12 bulls with matched metagenomic and host measurements. Rumen samples from the HS, HS + Se-L, and HS + Se-H groups were designated A1–A6, B1–B6, and C1–C6, respectively; A1–A4, B1–B4, and C1–C4 comprised the host-assay subsets.

### 2.3. Serum Biochemical and Endocrine Analyses

Serum total antioxidant capacity (T-AOC), superoxide dismutase (SOD) activity, glutathione peroxidase (GSH-Px) activity, and malondialdehyde (MDA) concentration were determined using commercial assay kits (Servicebio, Wuhan, China) according to the manufacturer’s instructions. T-AOC was determined using the ABTS method, SOD activity using the WST-1 method, GSH-Px activity using the DTNB method, and MDA concentration using the thiobarbituric acid (TBA) method. Absorbance was measured using a SpectraMax Plus 384 microplate reader (Molecular Devices, San Jose, CA, USA).

Serum concentrations of tumor necrosis factor-α (TNF-α), interleukin-1β (IL-1β), interleukin-6 (IL-6), testosterone, luteinizing hormone (LH), follicle-stimulating hormone (FSH), and inhibin B (INHB) were determined using bovine-specific ELISA kits (MLBIO, Shanghai, China) according to the manufacturer’s instructions. TNF-α, IL-1β, IL-6, LH, FSH, and INHB were determined using sandwich ELISA assays, whereas testosterone was determined using a competitive ELISA assay. Absorbance was measured at 450 nm using a SpectraMax Plus 384 microplate reader. Four biological replicates were analyzed per treatment group (*n* = 4).

### 2.4. Testicular Histology and TUNEL Assay

Testicular tissues from four bulls per treatment group were fixed in 4% paraformaldehyde (Servicebio, G1101) for 48 h, dehydrated through a graded ethanol series, cleared in xylene, embedded in paraffin, and sectioned at 4 μm. Sections were deparaffinized, rehydrated, and stained using a hematoxylin and eosin (H&E) staining kit (Servicebio, G1076).

For histological assessment, one evaluable large microscopic field containing multiple seminiferous tubules was selected per bull from an intact parenchymal area while avoiding tissue edges, folds, tears, and regions with obvious processing or staining artifacts. The same field-selection criteria were applied across animals. Spermatogenic status was assessed using a modified field-based application of the Johnsen scoring system [17]. Unlike the conventional approach in which multiple individual tubule cross-sections are scored separately, one overall score was assigned according to the predominant morphology and organization of the seminiferous epithelium across the tubules contained within the evaluated field. One histological score was obtained for each animal.

DNA fragmentation was assessed using a TMR-based one-step TUNEL apoptosis detection kit (Servicebio, G1502) according to the manufacturer’s instructions. Sections were treated with proteinase K and incubated with the TUNEL reaction mixture at 37 °C for 1 h, followed by nuclear counterstaining with DAPI. TUNEL-positive nuclei exhibited red fluorescence, whereas DAPI-stained nuclei exhibited blue fluorescence. One representative large microscopic field was analyzed per bull. The same field-selection criteria used for histological assessment were applied to the TUNEL analysis. TUNEL positivity was calculated as the number of TUNEL-positive nuclei divided by the total number of DAPI-stained nuclei, multiplied by 100%. Animal-level values were used for statistical analysis, with four bulls per treatment group. Across the evaluated fields, 1533–2591 DAPI-stained nuclei were counted per bull.

### 2.5. RNA Sequencing and Bioinformatics Analysis

Testicular RNA sequencing was performed using three biological replicates per treatment group (*n* = 3; nine samples in total). The samples Ctrl-1–3, Se-L-1–3, and Se-H-1–3 corresponded to animals A1–A3, B1–B3, and C1–C3, respectively. Total RNA was extracted from testicular tissues by the sequencing provider. RNA integrity and purity were assessed using agarose gel electrophoresis, a NanoDrop 2000 spectrophotometer (Thermo Fisher Scientific, Waltham, MA, USA), and an Agilent 2100 Bioanalyzer (Agilent Technologies, Santa Clara, CA, USA). Messenger RNA was enriched using mRNA capture beads and fragmented, followed by cDNA synthesis, end repair and A-tailing, adapter ligation, fragment selection, and PCR amplification. The resulting libraries were subjected to paired-end sequencing on an Illumina NovaSeq X Plus platform (Illumina, San Diego, CA, USA) by Omicsmaster Biotechnology Co., Ltd. (Guangzhou, China).

Raw reads were processed using fastp v0.18.0 to remove adapter-containing reads, reads containing more than 10% of unknown nucleotides, and reads in which more than 50% of bases had quality scores ≤ 20. Residual ribosomal RNA reads were removed using Bowtie2 v2.2.8. The remaining paired-end clean reads were mapped to the Bos taurus reference genome ARS-UCD1.3 (NCBI accession GCF_002263795.2) using HISAT2 v2.1.0. Mapped reads were assembled using StringTie v1.3.1, and gene-expression abundance was quantified using RSEM v1.2.19. Principal component analysis was performed using the gene-expression matrix to evaluate overall transcriptomic variation among samples. Because Se-H-3 separated from the other samples in the prespecified PCA, a descriptive sensitivity PCA was also generated after excluding Se-H-3 using log2(TPM + 1)-transformed expression values.

Differential-expression analysis was performed by the sequencing provider using DESeq2 with gene-level read counts as input. Candidate differentially expressed genes were initially screened using the provider-defined threshold of nominal *p* < 0.05 and |log_2_ fold change| > log_2_(1.5). For the present manuscript, gene-level FDR values were additionally considered, and genes meeting FDR < 0.05 together with the same fold-change threshold were classified as FDR-supported differentially expressed genes. The nominal-P screening results are reported separately in the Supplementary Materials.

KEGG gene set enrichment analysis was performed by the sequencing provider using expression-based gene rankings and the Signal2Noise metric. The exact KEGG release and permutation settings were not retained in the provider report. For the present manuscript, pathways with provider-reported FDR q < 0.05 were considered statistically supported. After examination of the complete enrichment results, six pathways directly related to cholesterol and steroid metabolism, transport, or secretion were selected for focused presentation; this selection was not prespecified. Complete results for all three contrasts are provided in the Supplementary Materials. Normalized enrichment score signs follow the provider’s contrast convention.

Because multiple transcript entries were annotated with the same gene symbol, all entries assigned to the five qPCR target symbols—*STAR*, *CYP11A1*, *CYP17A1*, *SCARB1*, and *LDLR*—were retained in the targeted heatmap. Gene or transcript identifiers were used to distinguish entries sharing the same symbol.

### 2.6. Quantitative Real-Time PCR

Total RNA was extracted from testicular tissues using RNA extraction reagent (Servicebio, G3013), and RNA concentration and purity were assessed using a NanoDrop 2000 spectrophotometer (Thermo Fisher Scientific, Waltham, MA, USA). Reverse transcription was performed using SweScript All-in-One RT SuperMix for qPCR with One-Step gDNA Remover (Servicebio, G3337). The reaction conditions were 25 °C for 5 min, 42 °C for 30 min, and 85 °C for 5 s.

Quantitative real-time PCR was performed on a CFX Connect Real-Time PCR Detection System (Bio-Rad Laboratories, Hercules, CA, USA) using 2× Universal Blue SYBR Green qPCR Master Mix (Servicebio, G3326). Each 15 μL reaction contained 7.5 μL of master mix, 1.5 μL of forward/reverse primer mixture (2.5 μM), 2.0 μL of cDNA, and 4.0 μL of nuclease-free water. The amplification program consisted of initial denaturation at 95 °C for 30 s, followed by 40 cycles of 95 °C for 15 s and 60 °C for 30 s. Melting-curve analysis was performed from 65 to 95 °C, with fluorescence recorded at 0.5 °C increments. Each sample was analyzed in technical triplicate.

Expression of *STAR*, *CYP11A1*, *CYP17A1*, *SCARB1*, and *LDLR* was normalized to *ACTB*. Technical-triplicate Cq values were averaged for each animal before ΔCq calculation. Relative expression was calculated using the 2^−ΔΔCq^ method [18], with A1 from the HS group as the common calibrator. Statistical comparisons used animal-level ΔCq values. Four biological replicates were included per treatment. Primer sequences, reference accession numbers, and amplicon lengths are listed in Table 2.

### 2.7. Western Blot Analysis

Total protein concentrations were determined using a BCA assay, and samples were adjusted according to their measured protein concentrations before denaturation. An equal amount of total protein (10 μg per lane) was loaded for SDS-PAGE. Proteins were separated at a constant voltage of 150 V and transferred onto PVDF membranes (Servicebio, G6047) by wet transfer at 350 mA for 30 min. Membranes were blocked with rapid blocking solution (Servicebio, G2052) for 10 min at room temperature and incubated overnight at 4 °C with primary antibodies against Bax (Servicebio, GB15007-100; 1:1000), Bcl-2 (Servicebio, GB154830-100; 1:1000), CYP17A1 (Servicebio, GB112095-100; 1:1000), or β-actin (Servicebio, GB12001; 1:2000). After washing with Tris-buffered saline containing 0.1% Tween-20 (TBST), membranes were incubated with HRP-conjugated goat anti-mouse IgG (Servicebio, GB23301) or goat anti-rabbit IgG (Servicebio, GB23303), as appropriate, at a dilution of 1:5000 for 30 min at room temperature. Protein bands were detected using enhanced chemiluminescence and imaged with an SCG-W3000 PLUS imaging system (Servicebio, Wuhan, China).

Band intensities were quantified using Chemiluminescence Imaging software v1.4.1 (Servicebio, Wuhan, China). For each animal, target-protein intensity was normalized to the corresponding β-actin intensity. The resulting ratios were then expressed relative to the mean ratio of the HS group. Four biological replicates were included per group.

### 2.8. Rumen Metagenomic Sequencing and Bioinformatics Analysis

Rumen fluid samples collected from six bulls per treatment group (*n* = 6 per group; *n* = 18 in total) were subjected to shotgun metagenomic sequencing. Microbial genomic DNA was extracted from each sample, and DNA quality was assessed before library construction. Sequencing libraries were constructed and subjected to paired-end 150-bp sequencing on an Illumina NovaSeq X Plus platform by Omicsmaster Biotechnology Co., Ltd. (Guangzhou, China).

Raw reads were quality-filtered using fastp v0.20.0 to remove low-quality reads and adapter contamination. Host-derived sequences were removed using Bowtie2 v2.3.5.1, and the resulting clean reads were assembled using MEGAHIT v1.2.9. Genes were predicted using MetaGeneMark v3.38 and clustered using CD-HIT v4.6 to construct a nonredundant gene catalog. Gene abundance was quantified using Salmon v1.10.3. Taxonomic annotation was performed at the read level using Kaiju v1.10.1 against the NCBI nonredundant protein database.

Functional annotation of the nonredundant gene catalog was performed against the KEGG database using DIAMOND v0.9.25. Functional abundance profiles were generated based on gene abundance and annotation. Differences in KEGG pathway abundance between treatment groups were evaluated using the pathway set available in the supplied functional-abundance table, with multiple-testing correction performed separately within each treatment contrast.

Microbiome–host association analyses were performed using the 12 bulls for which individually matched rumen metagenomic and host data were available. Genera with a mean relative abundance ≥ 0.1% and detected in at least nine of the 18 metagenomic samples were retained, resulting in 77 genera. These genera, together with three prespecified microbial functional pathways—riboflavin metabolism, ubiquinone and other terpenoid-quinone biosynthesis, and ABC transporters—were evaluated for associations with Johnsen score and TUNEL positivity. The same microbial features were separately evaluated against nine serum variables: T-AOC, SOD, GSH-Px, MDA, TNF-α, IL-6, testosterone, FSH, and INHB.

### 2.9. Statistical Analysis

Routine statistical analyses were performed using GraphPad Prism 8 (GraphPad Software, San Diego, CA, USA) and independently checked using NumPy v2.5.3, pandas v3.0.1, and SciPy v1.18.1. Individual bulls were treated as biological replicates. Data are presented as individual observations with mean ± SD unless otherwise indicated.

Serum, histological, and qPCR endpoints were analyzed using Kruskal–Wallis tests. When the omnibus test was significant, two-sided Dunn pairwise comparisons with tie correction were performed, and *p* values were adjusted using the Bonferroni procedure for the three pairwise comparisons within each endpoint. For qPCR, technical-triplicate Cq values were averaged for each animal, and statistical analyses were performed using unrounded animal-level ΔCq values. Western blot outcomes were analyzed using Welch’s one-way ANOVA followed by Games–Howell multiple-comparison tests. Because multiplicity was not controlled across the full set of serum endpoints, the serum analyses were interpreted as exploratory.

For rumen microbial diversity analyses, genus-level relative-abundance profiles were normalized within each sample. Shannon and Simpson diversity indices and the number of detected genera were calculated, and differences among treatment groups were evaluated using Kruskal–Wallis tests with Benjamini–Hochberg FDR correction across the three diversity measures. Bray–Curtis dissimilarities were used to assess community composition by principal coordinate analysis (PCoA) and permutational multivariate analysis of variance (PERMANOVA) with 9999 permutations. Homogeneity of multivariate dispersion was evaluated using PERMDISP. Pairwise PERMANOVA comparisons were adjusted for multiple testing using the Benjamini–Hochberg procedure.

Differences in KEGG pathway abundance were evaluated using two-sided Welch’s tests, with Benjamini–Hochberg FDR correction applied separately across the pathways included in each treatment contrast.

Spearman correlations were used to evaluate microbiome–host associations in the 12 individually matched bulls. The testis-focused analysis comprised 160 associations between 80 microbial features and Johnsen score or TUNEL positivity. To assess whether these associations were independent of treatment-group differences, a sensitivity analysis was additionally performed after adjusting ranks for treatment group. A separate family of 720 associations evaluated the same 80 microbial features against the nine serum endpoints. Multiple testing was controlled separately within the testis-focused, treatment-adjusted testis-focused, and serum association families using the Benjamini–Hochberg FDR procedure. FDR-adjusted *p* values (q) < 0.05 were considered statistically significant. These analyses were exploratory association tests and did not constitute formal mediation analysis.

## 3. Results

### 3.1. Serum Antioxidant, Inflammatory, and Reproductive Endocrine Responses

**Figure 1:**
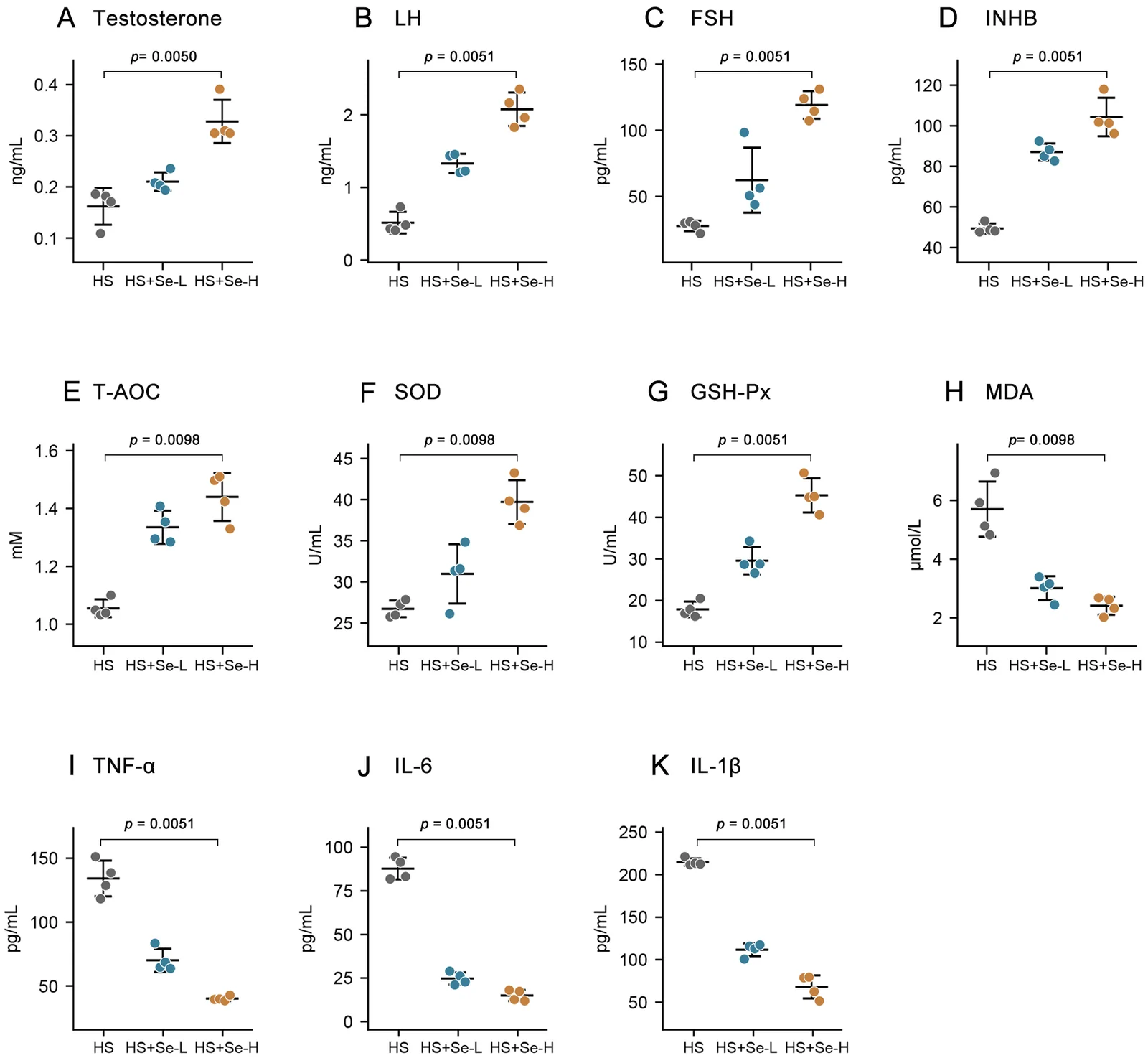
Serum responses to selenium-enriched malt supplementation in heat-exposed Simmental bulls. (**A**–**D**) Reproductive endocrine indices. (**E**–**H**) Antioxidant indices and MDA. (**I**–**K**) Inflammatory cytokines. Each point represents one bull; black marks indicate means ± SD (*n* = 4 per treatment). Brackets indicate comparisons between HS and HS + Se-H. Displayed *p* values are from two-sided Dunn tests following significant Kruskal–Wallis tests, with Bonferroni adjustment across the three pairwise comparisons within each endpoint. HS, heat-exposed control; HS + Se-L and HS + Se-H, low- and high-dose supplementation, respectively. Units are shown on each axis.

Serum reproductive hormone concentrations differed among groups (Figure 1A–D). Testosterone, LH, FSH, and INHB were higher in the HS + Se-H group than in the HS group (adjusted Dunn *p* < 0.01 for each endpoint). Mean concentrations were also numerically higher in the low-dose group, but its comparisons with HS did not meet the adjusted significance threshold.

The high-dose group had higher T-AOC, SOD, and GSH-Px values and lower MDA concentrations than the HS group (adjusted *p* < 0.01 for each endpoint; Figure 1E–H). Low-dose means changed in the same direction, although the corresponding pairwise comparisons were not significant after adjustment.

Serum TNF-α, IL-6, and IL-1β concentrations were lower in the HS + Se-H group than in the HS group (adjusted *p* < 0.01; Figure 1I–K). No other pairwise comparisons of these endpoints were significant after the specified correction. Exact omnibus and all pairwise adjusted *p* values for the serum, histological, and TUNEL endpoints are summarized in Table 3.

### 3.2. Testicular Histology and TUNEL Positivity

**Figure 2:**
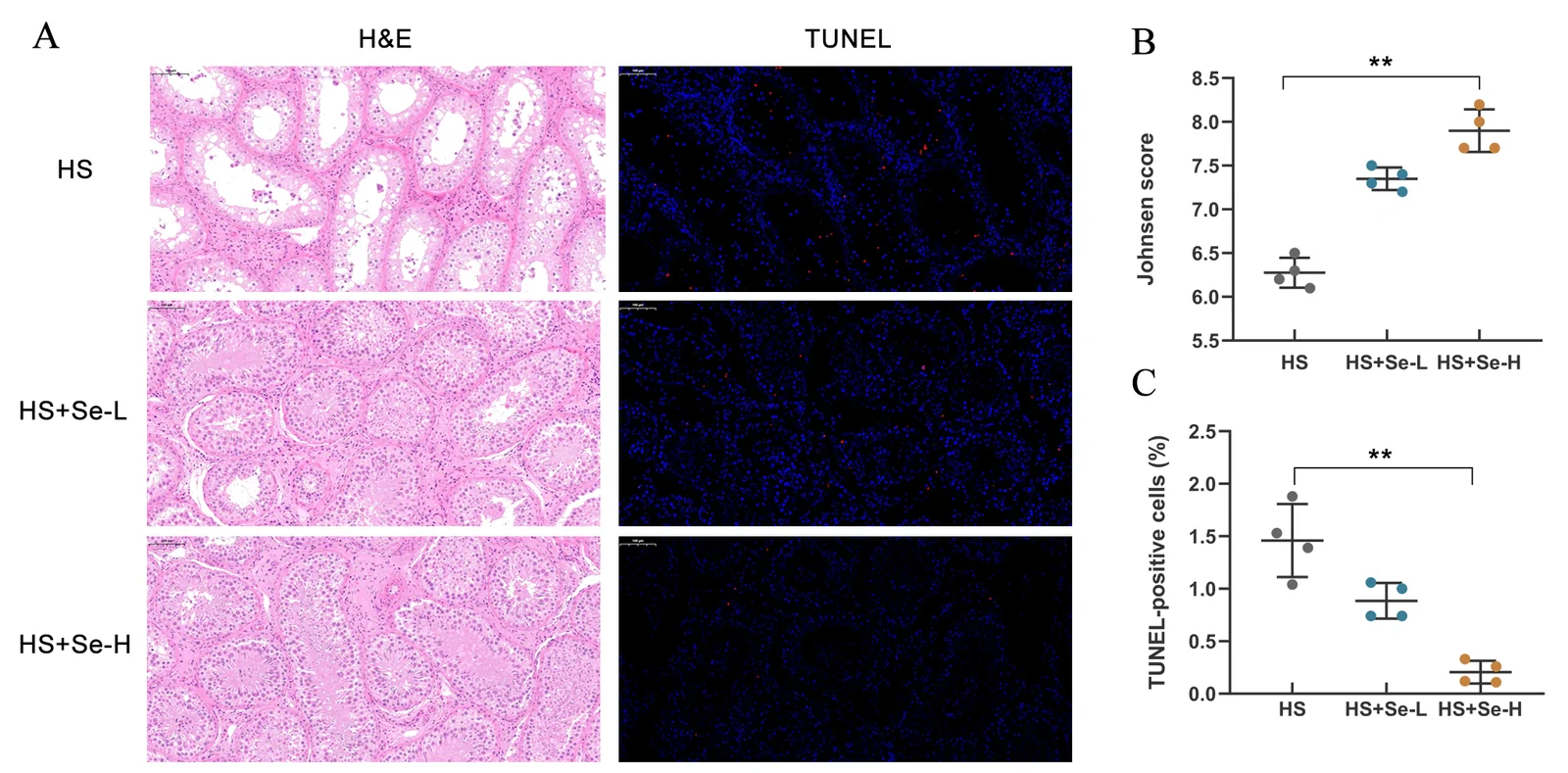
Testicular morphology and TUNEL positivity. (**A**) Representative H&E and TUNEL images. (**B**) Modified field-based Johnsen scores. (**C**) TUNEL-positive fractions. One large field containing multiple seminiferous tubules was evaluated per bull (*n* = 4 bulls per treatment). Quantitative data show individual observations and means ± SD. ** indicates adjusted *p* < 0.01 for HS + Se-H versus HS, using Kruskal–Wallis tests followed by Bonferroni-adjusted Dunn comparisons. Scale bars: 100 μm.

The evaluated H&E sections showed differences in seminiferous epithelial organization among groups, with comparatively better-preserved morphology in the high-dose group (Figure 2A). Mean Johnsen scores were 6.275, 7.350, and 7.900 in HS, HS + Se-L, and HS + Se-H, respectively. The high-dose group had a higher score than HS (adjusted Dunn *p* = 0.0050). The other pairwise comparisons were not significant.

Mean TUNEL-positive fractions were 1.460%, 0.885%, and 0.205% in HS, HS + Se-L, and HS + Se-H, respectively (Figure 2C). The high-dose group had lower TUNEL positivity than HS (adjusted Dunn *p* = 0.0070). Other pairwise comparisons were not significant. Both histological endpoints describe one evaluated field from each of four bulls per group.

### 3.3. Dose-Specific Transcriptomic Responses

**Figure 3:**
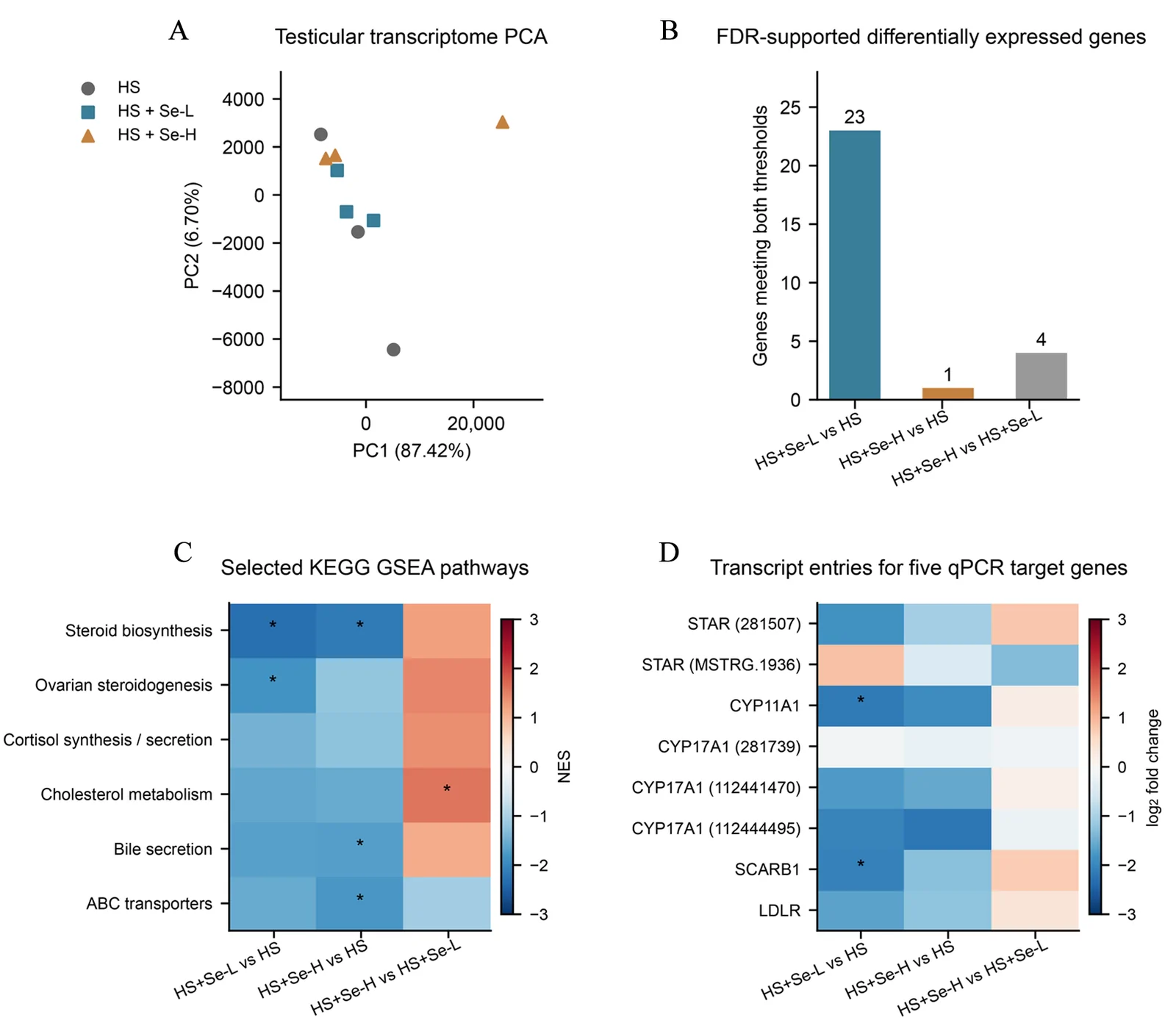
Testicular transcriptomic overview and targeted molecular context. (**A**) PCA coordinates for all nine samples. (**B**) Counts meeting gene-level FDR < 0.05 and |log_2_ fold change| > log_2_(1.5). (**C**) Provider-reported normalized enrichment scores (NES) for six pathways selected from the original analysis. (**D**) All supplied entries annotated with the five qPCR target symbols. Numbers in labels denote NCBI identifiers; MSTRG.1936 is the supplied assembled entry. Asterisks denote provider-reported GSEA q < 0.05 in panel (**C**) and gene-level FDR < 0.05 in panel (**D**); these panels represent different statistical families. RNA-seq *n* = 3 bulls per treatment.

Principal component analysis of the testicular RNA-seq expression profiles showed substantial between-sample variation, with Se-H-3 separated from the other samples along PC1 (Figure 3A). PC1 and PC2 explained 87.42% and 6.70% of the total variance, respectively. Using gene-level FDR < 0.05 together with the specified fold-change threshold, 23, 1, and 4 differentially expressed genes were identified in the HS + Se-L versus HS, HS + Se-H versus HS, and HS + Se-H versus HS + Se-L contrasts, respectively (Figure 3B). The corresponding nominal-*p* screening counts were 387, 281, and 197. In the descriptive sensitivity PCA, excluding Se-H-3, Se-H-1 and Se-H-2 remained close to one another, whereas the control and low-dose samples remained more dispersed; this analysis was used only to assess the PCA pattern and did not replace the prespecified nine-sample differential-expression or GSEA analyses.

KEGG GSEA identified 125 pathways with provider-reported q < 0.05 in at least one treatment contrast. Six pathways related to cholesterol and steroid metabolism, transport, or secretion are presented in Figure 3C. These six pathways were selected after examination of the complete enrichment results because of their direct relevance to the biological focus of the study and were not prespecified. Steroid biosynthesis showed negative NES values in the provider-labeled control-versus-low-dose (NES = −2.268, q < 0.0001) and control-versus-high-dose (NES = −2.111, q = 0.0036) contrasts, indicating enrichment toward the supplemented side under the provider’s contrast convention. These pathway-level findings were evaluated separately from gene-level differential expression.

Among the targeted gene entries, *SCARB1* and *CYP11A1* showed lower expression in HS + Se-L than in HS and met the gene-level FDR threshold (Figure 3D). *STAR* and *CYP17A1* were each represented by multiple supplied entries, which differed in direction or magnitude in some contrasts. All entries were therefore retained and distinguished using their gene or transcript identifiers.

### 3.4. qPCR Identified Lower Expression of Selected Targets at the Low Dose

**Figure 4:**
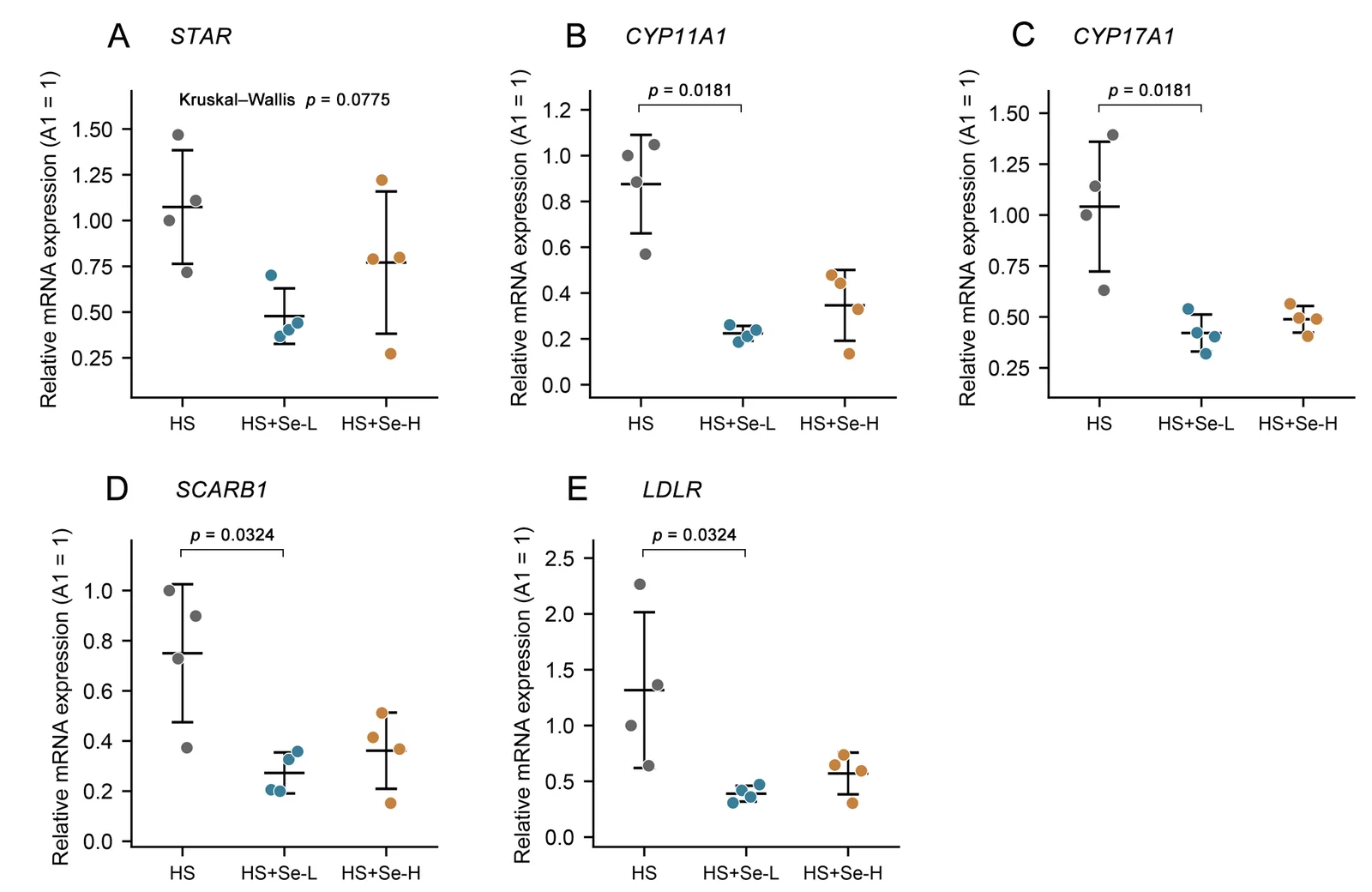
Relative mRNA expression of selected steroidogenesis- and cholesterol-related genes in testicular tissue. (**A**) *STAR*; (**B**) *CYP11A1*; (**C**) *CYP17A1*; (**D**) *SCARB1*; and (**E**) *LDLR*. Each point represents one bull; black marks indicate means ± SD (*n* = 4 per treatment). The value shown in panel (**A**) is from the Kruskal–Wallis test. Brackets in panels (**B**–**E**) indicate comparisons between HS and HS + Se-L. Displayed *p* values are from two-sided Dunn tests following significant Kruskal–Wallis tests, with Bonferroni adjustment across the three pairwise comparisons within each endpoint. Expression values were calculated relative to animal A1 from the HS group as the common calibrator.

qPCR showed lower *CYP11A1*, *CYP17A1*, *SCARB1*, and *LDLR* expression in HS + Se-L than in HS (Bonferroni-adjusted Dunn *p* = 0.0181, 0.0181, 0.0324, and 0.0324, respectively; Figure 4). *STAR* expression did not differ significantly among groups (Kruskal–Wallis *p* = 0.0775). Mean expression of the five targets was numerically lower in HS + Se-H than in HS, but no significant differences were detected between HS + Se-H and HS.

### 3.5. Testicular Bax, Bcl-2, and CYP17A1 Protein Abundance

**Figure 5:**
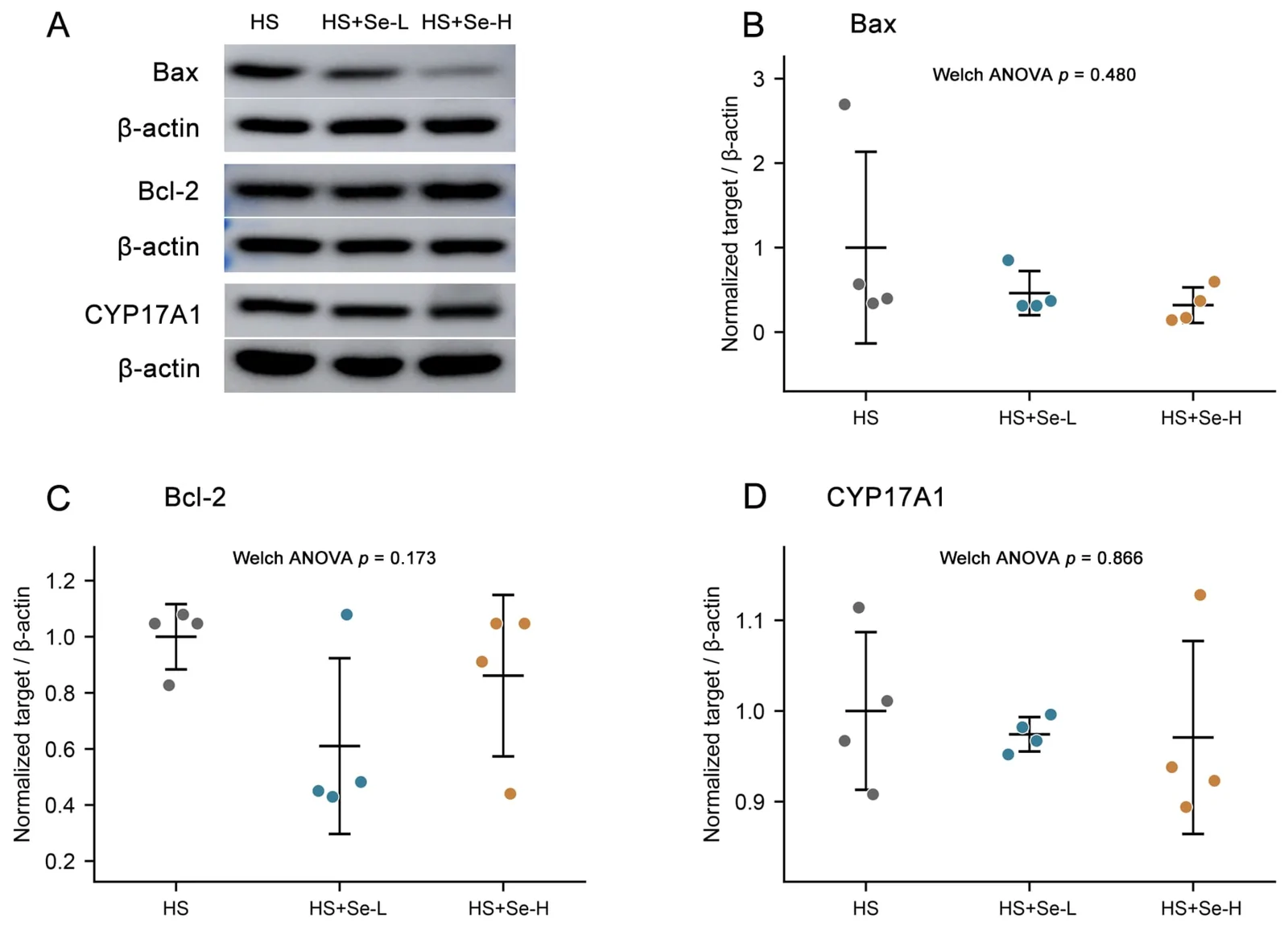
Testicular protein abundance. (**A**) Representative cropped Western blot bands for Bax, Bcl-2, CYP17A1, and their corresponding β-actin loading controls. Full membrane images and lane assignments are provided in Figure S6. (**B**–**D**) Corresponding normalized protein measurements. Target/β-actin ratios were expressed relative to the mean of the HS group. Data show individual values and means ± SD (*n* = 4 bulls per treatment). Welch’s ANOVA followed by Games–Howell comparisons detected no significant group differences.

Western blot analysis showed no significant differences in testicular Bax, Bcl-2, or CYP17A1 protein abundance among treatment groups (Welch’s ANOVA *p* = 0.480, 0.173, and 0.866, respectively; Figure 5). None of the Games–Howell pairwise comparisons was significant. Individual values and normalized group means are presented in Figure 5, and uncropped membrane images with complete lane assignments are provided in Figure S6.

### 3.6. Rumen Microbial Composition and Functional Profiles Differed Among Treatments

**Figure 6:**
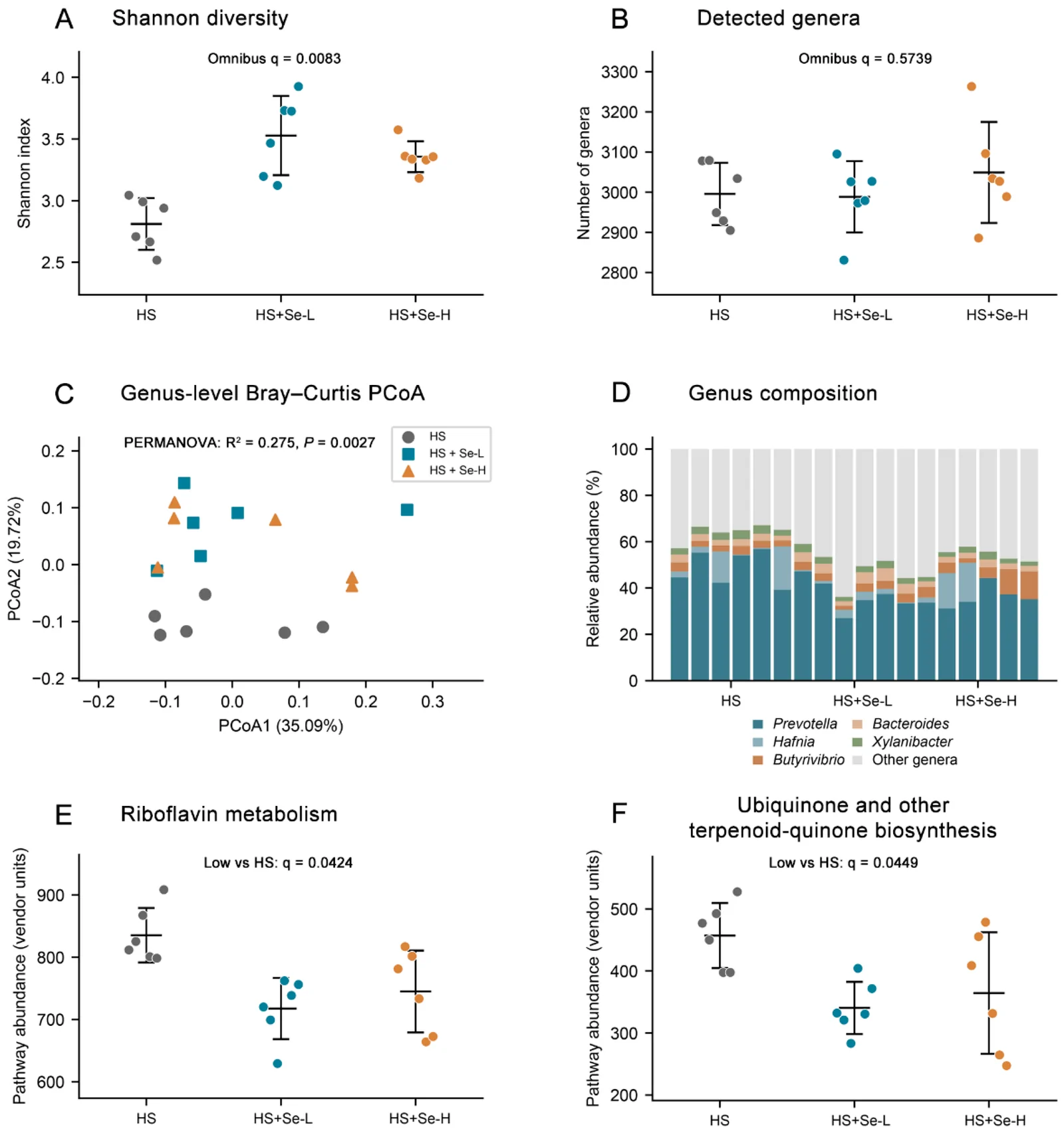
Rumen microbial diversity, community composition, and targeted functional profiles. (**A**) Shannon diversity. (**B**) Detected genera. (**C**) Bray–Curtis PCoA. (**D**) Five most abundant genera ranked by mean abundance across all 18 samples; remaining genera are pooled for display only. Bars represent A1–A6, B1–B6, and C1–C6 within their respective groups. (**E**,**F**) Two originally targeted KEGG pathways. Points show individual bulls and means ± SD where displayed (*n* = 6 per group). Alpha-diversity q values are corrected across three omnibus tests. PERMANOVA used 9999 permutations. PCoA percentages use positive eigenvalues. Pathway q values were derived from two-sided Welch’s tests with FDR correction across the pathways included in the HS + Se-L versus HS contrast. Pathway abundance retains the supplied vendor scale. Complete pathway-level and diversity statistics, including PERMDISP results, are provided in Table S3. HS, gray; Se-L, blue; Se-H, orange in panels (**A**–**C**,**E**,**F**).

Genus-level microbial diversity differed among treatment groups. Shannon diversity showed a significant overall difference after FDR correction (q = 0.0083), whereas the number of detected genera did not differ significantly (q = 0.5739; Figure 6A,B). Simpson diversity also differed among groups (q = 0.0214; Figure S4A). Relative-abundance profiles provided a descriptive overview of the dominant genera across all 18 bulls (Figure 6D).

Bray–Curtis community composition differed significantly among groups (PERMANOVA, R^2^ = 0.275, *p* = 0.0027; Figure 6C). This difference was not accompanied by a significant difference in multivariate dispersion (PERMDISP, *p* = 0.7227; Figure S4B), indicating that the PERMANOVA result was unlikely to be explained solely by differences in within-group dispersion.

In the pathway-level analysis used for the present manuscript, FDR-supported differences were detected in the HS + Se-L versus HS contrast. The main-text display focuses on two cofactor-related pathways selected post hoc from this analysis and is interpreted as exploratory. Riboflavin metabolism and ubiquinone and other terpenoid-quinone biosynthesis showed lower relative abundance in HS + Se-L than in HS (q = 0.0424 and 0.0449, respectively; Figure 6E,F). Complete pathway-level results for all treatment contrasts, based on the annotation and statistical workflow used here, are provided in Table S3.

### 3.7. Exploratory Microbiome–Testis Associations

To explore potential relationships between rumen microbial features and testicular phenotypes, 160 associations between 80 microbial features and two direct testicular endpoints (Johnsen score and TUNEL positivity) were evaluated in the 12 individually matched bulls using Spearman correlations.

**Figure 7:**
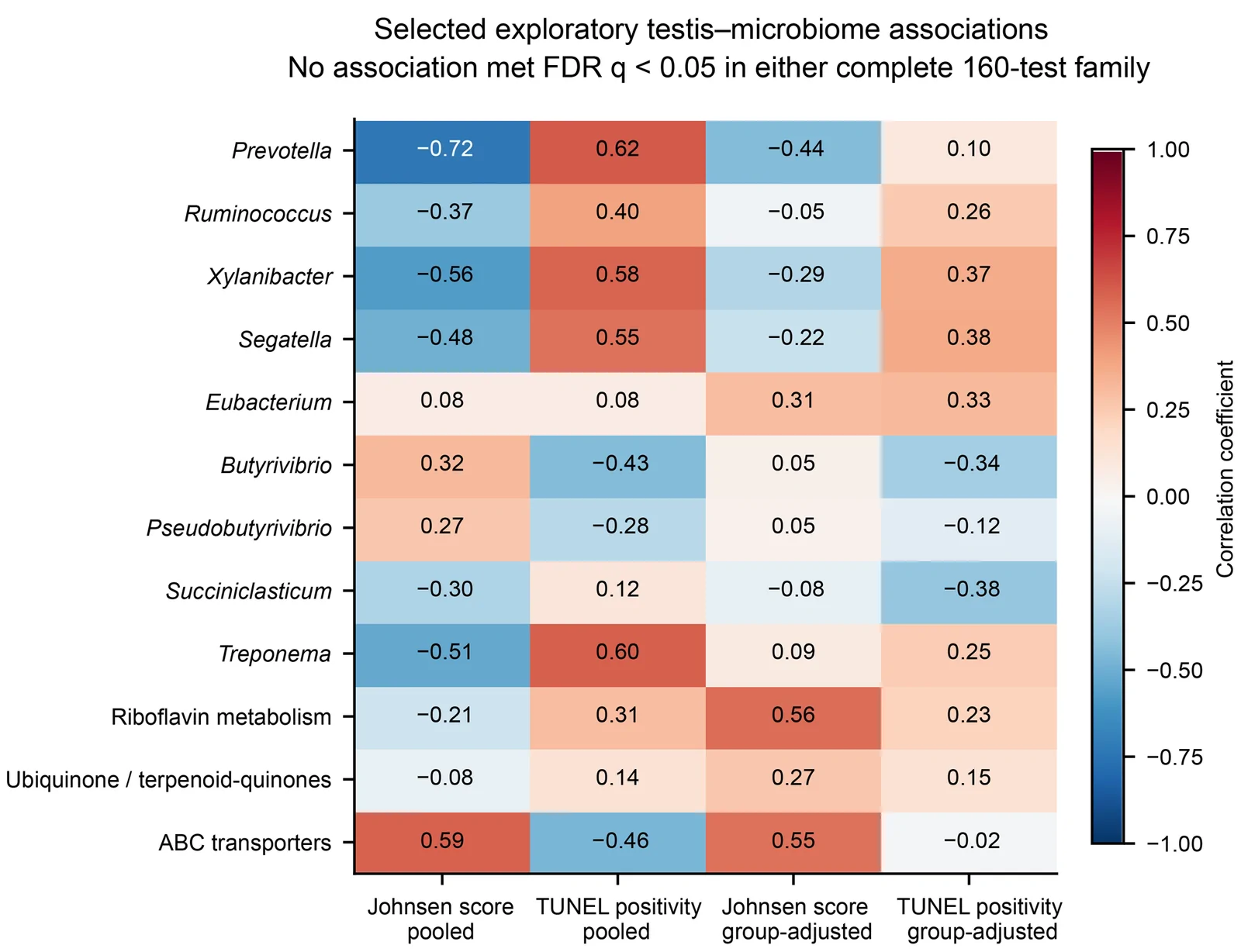
Nine genera highlighted in the original analysis and three targeted KEGG pathways are displayed. Values represent pooled Spearman correlation coefficients or correlations between ranks residualized for treatment group. Analyses included 12 individually matched bulls, with four bulls per treatment. Pooled tests used 9999 permutations, whereas treatment-adjusted tests used within-group permutations. FDR correction was applied separately to the complete 160-test testis family, the 160-test treatment-adjusted testis family, and the 720-test serum family. No association in any family met FDR q < 0.05. Complete results are provided in Table S4.

Several nominal correlations were observed, but none of the 160 testis-focused associations remained significant after Benjamini–Hochberg FDR correction. No testis-focused association remained significant in the treatment-adjusted sensitivity analysis (Figure 7). Likewise, none of the 720 associations between the same microbial features and the nine serum endpoints survived FDR correction.

## 4. Discussion

### 4.1. Systemic Antioxidant, Inflammatory, and Reproductive Endocrine Responses

The high-dose group showed coordinated differences in serum antioxidant, inflammatory, and reproductive endocrine measurements, together with higher Johnsen scores and lower TUNEL positivity. Selenium-dependent redox regulation provides a biologically plausible context for these responses [5,7]. Related systemic responses have been reported for hydroxy-selenomethionine in heat-exposed dairy cows [10] and for selenium-, zinc-, and copper-containing supplements in steers [19,20], although those interventions differ from selenium-enriched malt.

The lower circulating inflammatory cytokine concentrations accompanied higher antioxidant indices, consistent with interactions between redox balance and inflammatory signaling during heat exposure [5,19]. Because local testicular redox and inflammatory pathways were not measured, the serum findings are best interpreted as a systemic response that occurred alongside the tissue changes.

Higher circulating testosterone, LH, FSH, and INHB in the high-dose group indicate a more favorable reproductive endocrine profile under the conditions studied. Selenium has established roles in male reproductive physiology [5,21,22]. These hormone data complement the histological findings, although semen quality and fertility were not evaluated and the absence of a thermoneutral group prevents estimation of recovery toward non-stressed values.

### 4.2. Selenium-Enriched Malt Supplementation Was Associated with More Favorable Testicular Histology

The histological findings provide the most direct testicular evidence in this study. Higher modified Johnsen scores and better-preserved seminiferous epithelial organization in the high-dose group indicate a more favorable spermatogenic tissue state. Thermal injury can affect germ, Sertoli, and Leydig cells through oxidative and other cellular processes [2,23,24]. The use of one representative field per animal limits tissue coverage, although the animal-level analysis identified a difference between HS + Se-H and HS.

Lower TUNEL positivity in the high-dose group indicates less detectable DNA fragmentation in the evaluated fields and supports the histological result. Bax and Bcl-2 protein abundance did not differ significantly, showing that the tissue response was not accompanied by detectable changes in these proteins. TUNEL staining measures DNA fragmentation and does not identify the responsible cell-death pathway; therefore, the tissue findings should not be attributed specifically to a Bax/Bcl-2-related mechanism.

The unchanged CYP17A1 protein abundance also contrasts with the higher circulating reproductive hormone concentrations at the high dose. This divergence emphasizes that the tissue, endocrine, transcript, and protein measurements capture different levels of regulation. The consistent histological and TUNEL findings remain the principal evidence for a testicular response.

### 4.3. Dose-Specific Transcriptional Findings

The transcriptomic results differed markedly by dose and by analytical level. Gene-level FDR control reduced the nominal screening lists to 23, 1, and 4 supported genes across the three contrasts, whereas GSEA identified cholesterol- and steroid-related gene sets [25,26]. Pathway-level enrichment can detect coordinated changes that are not represented by many individually significant genes, but it does not establish activation of every member of a pathway. The between-sample variation and three-bull RNA-seq subset should therefore be considered when interpreting treatment patterns.

The low-dose qPCR results were directionally consistent with lower expression of several cholesterol-handling and steroidogenesis-related targets, including *CYP11A1*, *CYP17A1*, *SCARB1*, and *LDLR*. By contrast, the clearest histological and endocrine responses occurred at the high dose, and CYP17A1 protein abundance was unchanged. These findings indicate a dose-specific molecular response rather than uniform activation of steroidogenesis.

Steroidogenesis depends on substrate availability, endocrine stimulation, enzyme activity, and post-transcriptional regulation as well as mRNA abundance [25,26]. These regulatory levels may contribute to the observed divergence, but they were not directly tested. Repeated *STAR* and *CYP17A1* annotations in the RNA-seq output further limit a simple one-to-one validation narrative across sequencing and targeted assays.

The contrast between the high-dose tissue response and the larger low-dose transcriptional response further highlights the dose-specific nature of the findings. The current molecular data cannot define an optimal dose or establish a single mechanistic sequence. Future studies using larger matched molecular datasets and direct measurements of steroid production could help clarify these dose-dependent relationships.

### 4.4. Rumen Microbial Composition and Functional Potential

Selenium-enriched malt supplementation was accompanied by differences in rumen microbial diversity and community composition, consistent with previous reports of ruminal responses to selenium-containing diets [13,14]. The significant PERMANOVA result occurred without a detectable difference in multivariate dispersion, supporting a difference in community composition among treatments. Although greater microbial diversity should not itself be interpreted as improved rumen function, these results demonstrate a community-level microbial response to supplementation.

In the pathway-level analysis used for the present manuscript, FDR-supported differences were detected in the HS + Se-L versus HS contrast. The two cofactor-related pathways highlighted in the main figure, riboflavin metabolism and ubiquinone and other terpenoid-quinone biosynthesis, showed lower relative abundance in HS + Se-L than in HS. Riboflavin supports flavin-dependent reactions, and dietary riboflavin can influence ruminal microbial and fermentation traits [27]. Because metagenomic abundance represents functional potential, corresponding metabolite or fermentation measurements would be required to determine the physiological consequences of these pathway differences.

The microbial responses were dose-dependent and did not parallel the testicular histological pattern, which was clearest at the high dose. This difference in dose-response patterns argues against interpreting the microbial and testicular findings as a single linear pathway. The matched association analysis was therefore used to explore whether specific microbial features covaried with direct testicular endpoints.

No testis-focused microbial association remained significant after FDR correction, either before or after adjustment for treatment group. The serum association family produced the same overall result. Thus, the concurrent rumen and host responses did not identify a specific microbial feature linking supplementation to the testicular phenotype. These exploratory association tests were not formal mediation models, and the present results do not resolve whether rumen microbial changes contributed to the testicular responses. Experimental microbiota-transfer studies in mice have suggested that gut microbial alterations can influence sperm quality and spermatogenesis [28]. Larger longitudinal studies with prespecified microbial targets and formal mediation models could examine this possibility in cattle.

### 4.5. Study Strengths and Limitations

A strength of this study is the integration of direct testicular histology and TUNEL measurements with serum, molecular, and rumen metagenomic data. The use of individually matched animals also allowed microbial associations to be evaluated against direct testicular endpoints rather than serum measurements alone.

The study also has important limitations. Host assays used four bulls per treatment, RNA-seq used three per treatment, and histological quantification covered one field per animal. These sample sizes limit precision and statistical power, particularly for the molecular and association analyses. Because no thermoneutral control was included and the total selenium concentration of the basal diet was not chemically quantified, the findings are interpreted as treatment-associated responses under heat-exposure conditions and do not define recovery to thermoneutral status or an optimal selenium dose.

Semen quality and fertility were not measured in the study animals, so the findings concern testicular tissue integrity and endocrine status rather than demonstrated fertility improvement. The supplementation levels are therefore reported as target supplemental selenium levels, and potential effects of the malt carrier cannot be completely separated from those of selenium. Microbial functional profiles were not paired with rumen metabolites or fermentation outcomes.

Within these limitations, the convergence of serum and direct testicular findings supports a nutritional response to selenium-enriched malt under field heat exposure. The dose-specific transcriptomic and microbial findings provide priorities for further confirmatory studies.

## 5. Conclusions

Selenium-enriched malt supplementation, particularly the regimen targeting 0.5 mg supplemental Se/kg dietary DM, was associated with a more favorable systemic antioxidant, inflammatory, and reproductive endocrine profile, higher Johnsen scores, and lower testicular TUNEL positivity in heat-exposed Simmental bulls. Transcriptomic and qPCR responses were dose-specific, whereas Bax, Bcl-2, and CYP17A1 protein abundance did not differ among treatments. Supplementation was also accompanied by differences in rumen microbial diversity, community composition, and selected functional profiles. These findings characterize systemic, testicular, molecular, and microbial responses under field heat exposure and support further evaluation of selenium-enriched malt in cattle nutrition; they do not define an optimal selenium dose or establish microbial mediation of the testicular responses.

## Figures and Tables

**Table 2:** Primer sequences used for quantitative real-time PCR.

Gene	Forward Primer (5′–3′)	Reverse Primer (5′–3′)	Reference Accession	Amplicon (bp)
*STAR*	GGACGAGGTGCTGAGTAAAGTGA	TTCCAATCTTCTGCAGGACCTT	NM_174189.4	170
*CYP11A1*	ACCGATATTATCAGAAACCCATTG	CAAAGGCAAAGTGAAACAGGTC	NM_176644.2	234
*CYP17A1*	GCCCCATCTATTCCTTTCGTTT	AATGCCCTTTTGGTTGTCTGA	NM_174304.3	155
*SCARB1*	TTCAGGCACAAGAGCAACATCA	ACGCACTCAAGACCAGGATG	NM_174597.2	145
*LDLR*	TTGGACGGATGTTATCAACGAAG	TCTCACACCAGTTCACCCCTCT	NM_001166530.1	149
*ACTB*	GCGGCATTCACGAAACTACC	ACTCCTGCTTGCTGATCCAC	NM_173979.3	270

Abbreviations: STAR, steroidogenic acute regulatory protein; CYP11A1, cytochrome P450 family 11 subfamily A member 1; CYP17A1, cytochrome P450 family 17 subfamily A member 1; SCARB1, scavenger receptor class B member 1; LDLR, low-density lipoprotein receptor; ACTB, beta-actin.

**Table 3:** Summary of serum, histological, and TUNEL outcomes.

Outcome	HS	HS + Se-L	HS + Se-H	Omnibus *p*	Dunn *p*: HS–L/HS–H/L–H
Testosterone (ng/mL)	0.162 ± 0.036	0.210 ± 0.018	0.328 ± 0.042	0.0072	0.3481/0.0050/0.3481
LH (ng/mL)	0.514 ± 0.148	1.330 ± 0.133	2.076 ± 0.230	0.0073	0.3500/0.0051/0.3500
FSH (pg/mL)	27.7 ± 4.0	62.3 ± 24.6	119.2 ± 10.5	0.0073	0.3500/0.0051/0.3500
INHB (pg/mL)	49.4 ± 2.5	87.1 ± 4.3	104.3 ± 9.5	0.0073	0.3500/0.0051/0.3500
T-AOC (mM)	1.055 ± 0.031	1.335 ± 0.057	1.440 ± 0.083	0.0125	0.2327/0.0098/0.7179
SOD (U/mL)	26.72 ± 1.01	30.98 ± 3.61	39.72 ± 2.66	0.0125	0.7179/0.0098/0.2327
GSH-Px (U/mL)	17.86 ± 1.88	29.57 ± 3.30	45.27 ± 4.12	0.0073	0.3500/0.0051/0.3500
MDA (μmol/L)	5.70 ± 0.94	3.01 ± 0.41	2.41 ± 0.31	0.0125	0.2327/0.0098/0.7179
TNF-α (pg/mL)	134.2 ± 14.1	70.1 ± 9.1	40.1 ± 1.9	0.0073	0.3500/0.0051/0.3500
IL-6 (pg/mL)	87.8 ± 6.2	24.8 ± 3.5	15.0 ± 3.2	0.0073	0.3500/0.0051/0.3500
IL-1β (pg/mL)	214.7 ± 4.3	111.7 ± 7.5	68.0 ± 13.6	0.0073	0.3500/0.0051/0.3500
Modified Johnsen score	6.28 ± 0.17	7.35 ± 0.13	7.90 ± 0.24	0.0072	0.3481/0.0050/0.3481
TUNEL-positive fraction (%)	1.460 ± 0.348	0.885 ± 0.169	0.205 ± 0.108	0.0096	0.5072/0.0070/0.2848

Values are mean ± SD (*n* = 4 bulls per group). Omnibus *p* values are from Kruskal–Wallis tests. Dunn *p* values are Bonferroni-adjusted and are listed in the order HS versus HS + Se-L, HS versus HS + Se-H, and HS + Se-L versus HS + Se-H.

## Data Availability

The raw testicular RNA-seq and rumen metagenomic data have been deposited in the Genome Sequence Archive (GSA) of the National Genomics Data Center, China National Center for Bioinformation, under BioProject PRJCA077512, with accession numbers CRA052327 and CRA052485, respectively. Other data supporting the findings of this study are provided within the article and its Supplementary Materials or are available from the corresponding author upon reasonable request.

## Supplementary Materials

The following supporting information can be downloaded at: https://www.mdpi.com/article/10.3390/ani16213485/s1, Figure S1, Transcriptomic PCA and gene-level FDR volcano plots; Figure S2, Complete significant KEGG GSEA overview; Figure S3, Provider steroid-biosynthesis enrichment curve; Figure S4, Additional rumen microbial diversity and dispersion analyses; Figure S5, Selected exploratory microbiome–testis association scatterplots; Figure S6, Uncropped Western blot membrane images supporting Figure 5; Table S1, Complete RNA-seq differential-expression results; Table S2, Complete KEGG GSEA results; Table S3, Rumen diversity, PCoA, dispersion, and KEGG pathway statistics; Table S4, Complete microbiome–host association results. Tables S1–S4 are provided as contrast- or analysis-specific data files in a single ZIP archive.
