# Supplementary material for: Testicular and Rumen Microbial Responses to Selenium-Enriched Malt in Heat-Exposed Simmental Bulls
Source: Animals (Basel). 2026 Nov 4;16(21):3485. doi: 10.3390/ani16213485 (PMC13761496; doi:10.3390/ani16213485)
Supplement: Supplementary file 1 [file animals-16-03485-s001.zip › animals-4665074-supplementary--1st revision.pdf]

Supplementary Materials

Testicular and Rumen Microbial Responses to Selenium-Enriched Malt in Heat-Exposed Simmental Bulls

Supplementary Methods

Gene-level differential-expression tables were screened using the supplied P values, FDR values, and fold changes. Nominal-P screening used $P < 0.05$ and $|\log 2 \text{ fold change}| > \log 2(1.5)$; the manuscript classification additionally required gene-level $FDR < 0.05$. The provider DESeq2 model was not refitted. GSEA q values are provider-reported and constitute a separate statistical family. A reported GSEA q value of zero reflects the provider output and should not be interpreted as a probability of exactly zero.

Rumen community analyses used the 18 genus-abundance profiles. Bray–Curtis PERMANOVA used 9,999 permutations, and dispersion was assessed using PERMDISP. Matched microbiome–host analyses included 12 bulls. Testis-focused and serum families comprised 160 and 720 tests, respectively. Pooled association tests used 9,999 permutations, and the treatment-adjusted sensitivity analysis enumerated 13,824 within-group permutations. Benjamini–Hochberg correction was applied separately to each complete association family.

Supplementary Tables

Complete results for Tables S1–S4 are provided as separate machine-readable CSV files in the accompanying Supplementary_Tables_S1-S4.zip archive, submitted alongside this Supplementary Materials document. The archive contains analysis- and contrast-specific files labeled Tables S1A–S1C, S2A–S2C, S3A–S3F, and S4A–S4B. The files are provided separately to retain all result rows and machine-readable numeric precision.

Table	Content	Scope
S1	RNA-seq differential-expression results	23,563 entries per contrast; three contrasts
S2	Complete KEGG GSEA results	All supplied gene sets and three contrasts
S3	Rumen diversity, PCoA, dispersion and KEGG comparisons	18 bulls; complete supplied pathway-level results for each treatment contrast
S4	Complete microbiome–host associations	160 testis-focused and 720 serum associations; treatment-adjusted testis results included

Table S1 summary

Contrast	Nominal-P screening	FDR-supported DEGs
Low dose vs HS	387	23
High dose vs HS	281	1
High dose vs low dose	197	4

Figure S1 Transcriptomic PCA and gene-level FDR volcano plots

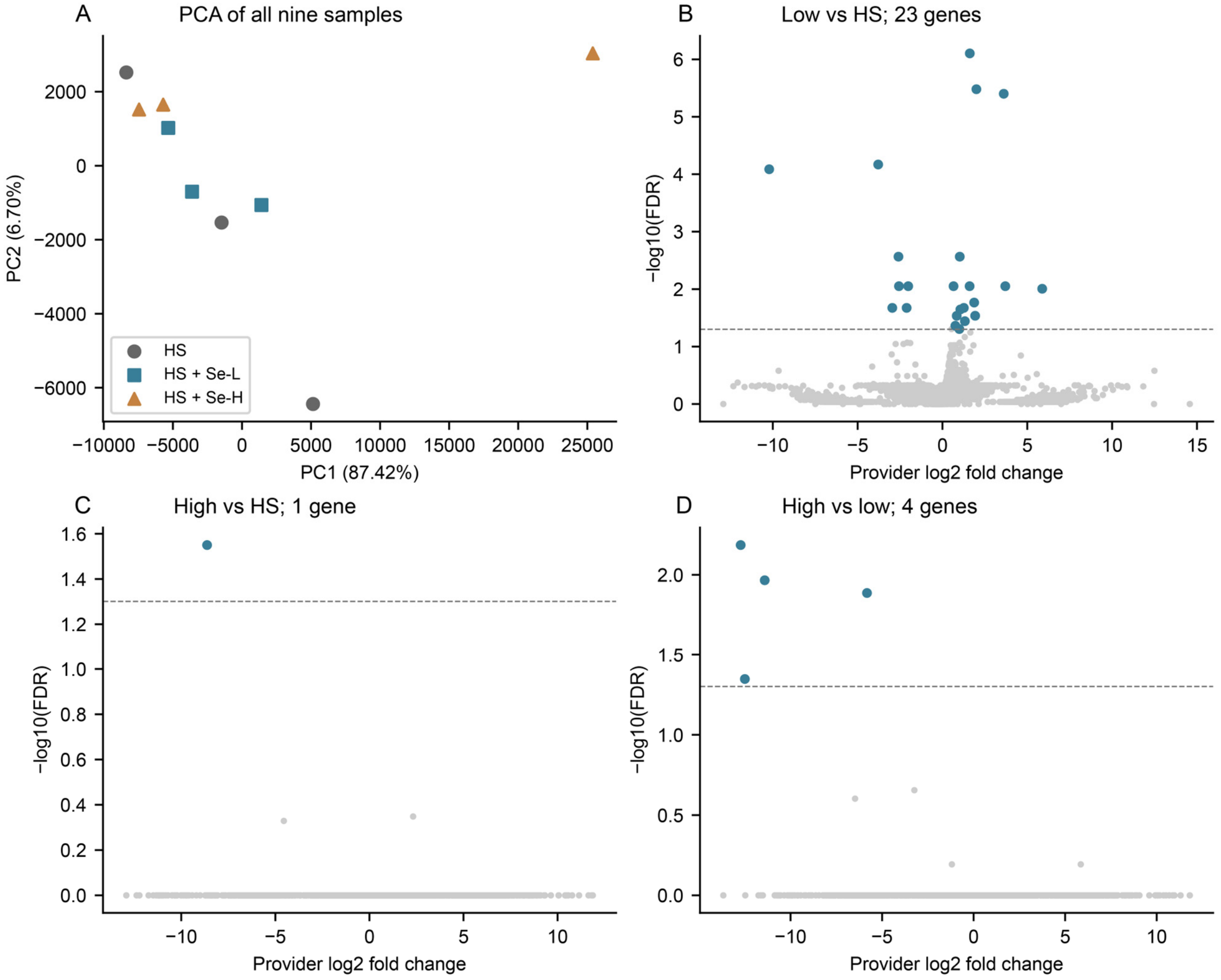

Figure S1. All nine RNA-seq samples were retained. Colored points meet FDR < 0.05 and |log2 fold change| > log2(1.5). Nominal-P screening results are reported separately in Table S1.

Figure S2 (1/6) Complete significant KEGG GSEA overview

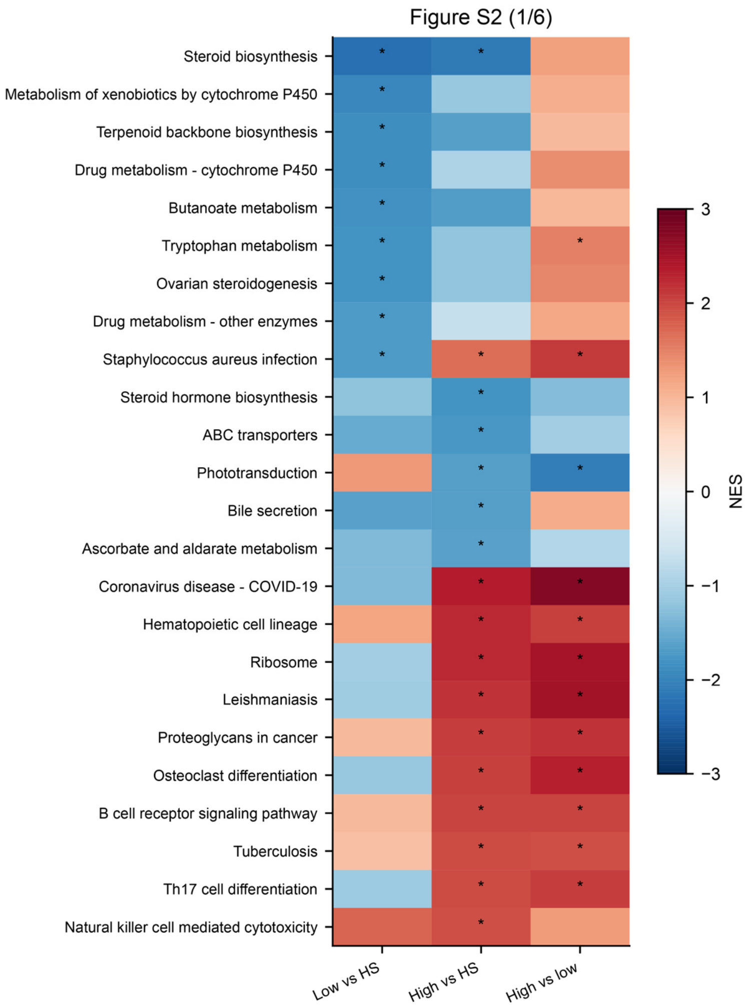

Figure S2 (1/6). Part 1 of the union of 125 pathways with provider-reported q < 0.05 in at least one contrast. Color represents NES; * indicates provider-reported q < 0.05. Complete results are in Table S2. NES follows the provider contrast convention and does not establish pathway activation.

Figure S2 (2/6) Complete significant KEGG GSEA overview

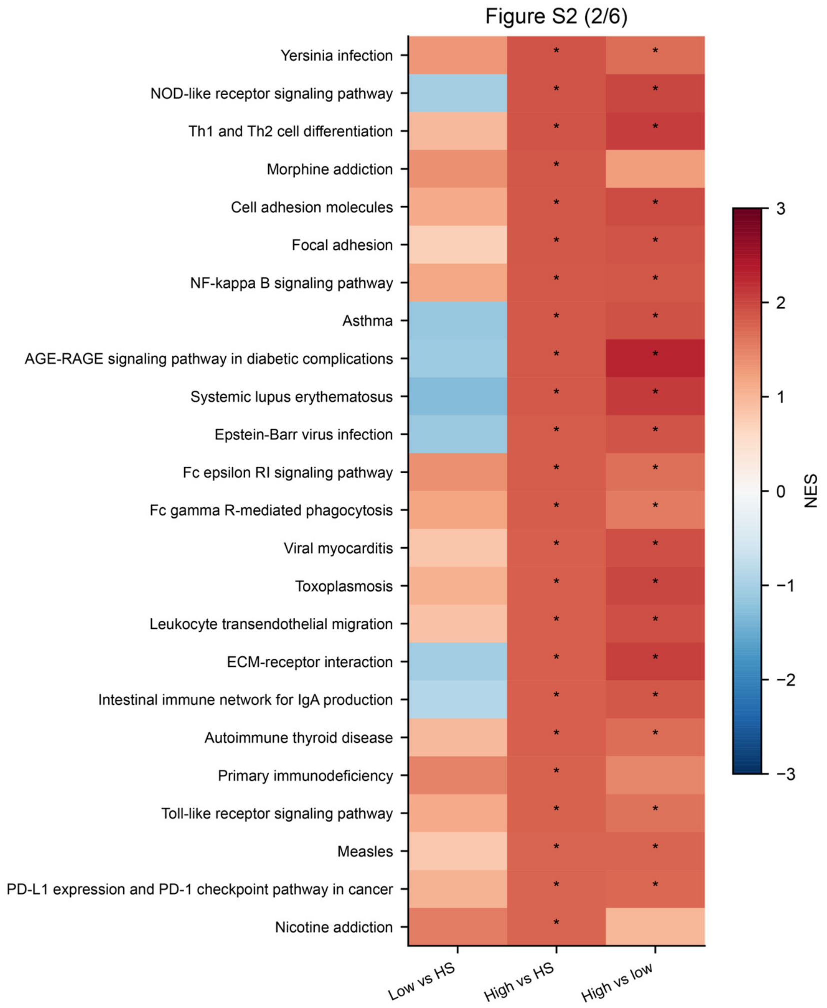

Figure S2 (2/6). Part 2 of the union of 125 pathways with provider-reported q < 0.05 in at least one contrast. Color represents NES; * indicates provider-reported q < 0.05. Complete results are in Table S2. NES follows the provider contrast convention and does not establish pathway activation.

Figure S2 (3/6) Complete significant KEGG GSEA overview

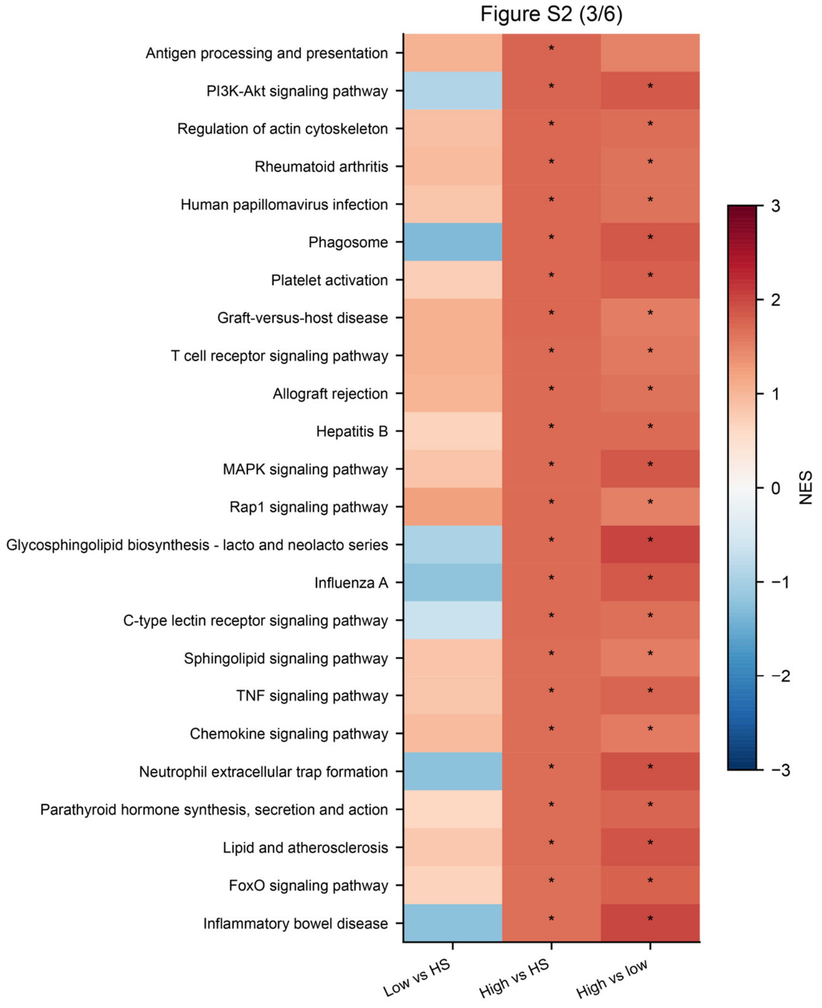

Figure S2 (3/6). Part 3 of the union of 125 pathways with provider-reported q < 0.05 in at least one contrast. Color represents NES; * indicates provider-reported q < 0.05. Complete results are in Table S2. NES follows the provider contrast convention and does not establish pathway activation.

Figure S2 (4/6) Complete significant KEGG GSEA overview

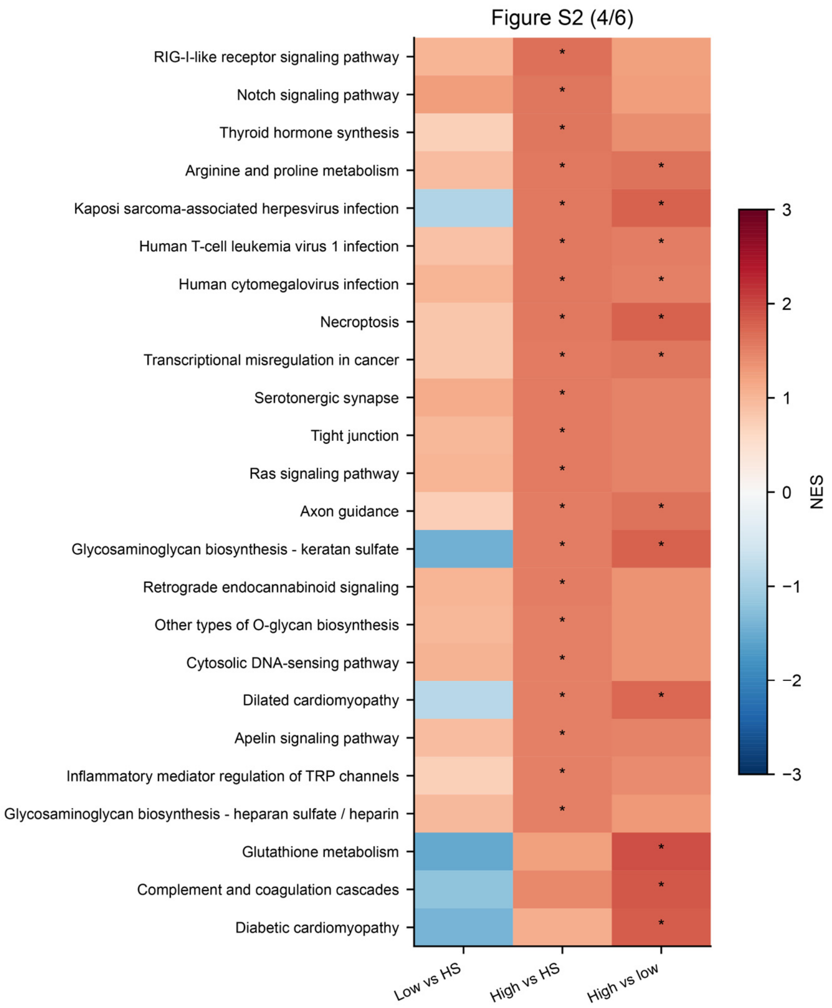

Figure S2 (4/6). Part 4 of the union of 125 pathways with provider-reported q < 0.05 in at least one contrast. Color represents NES; * indicates provider-reported q < 0.05. Complete results are in Table S2. NES follows the provider contrast convention and does not establish pathway activation.

Figure S2 (5/6) Complete significant KEGG GSEA overview

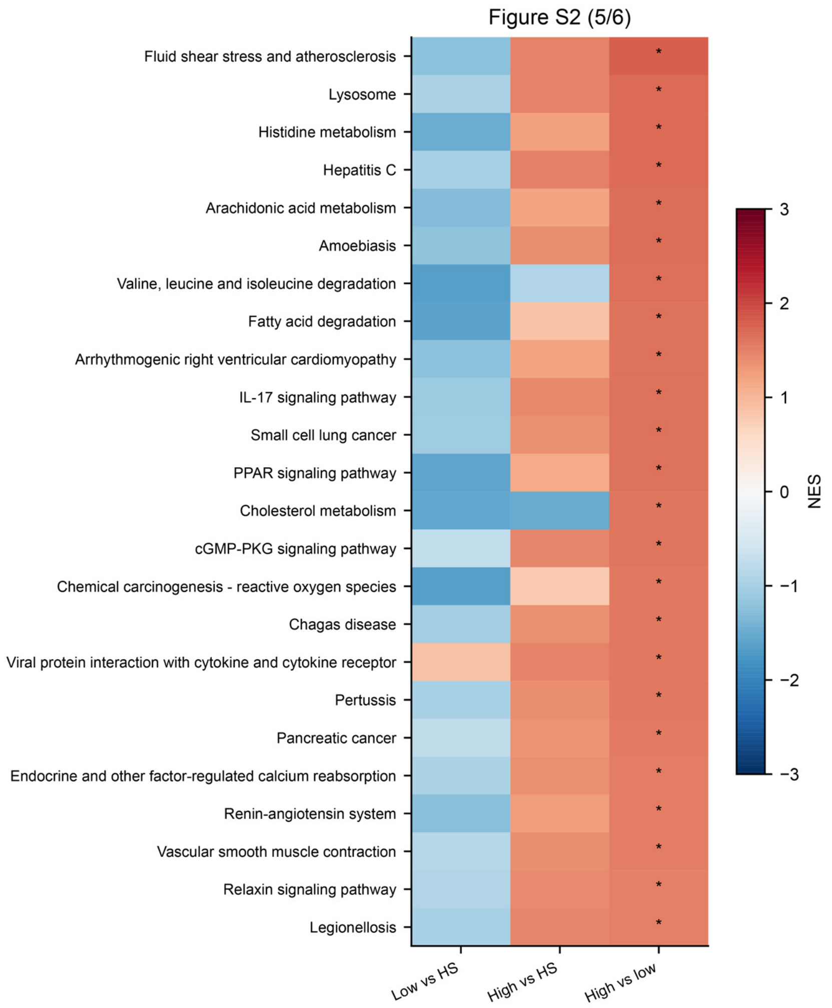

Figure S2 (5/6). Part 5 of the union of 125 pathways with provider-reported q < 0.05 in at least one contrast. Color represents NES; * indicates provider-reported q < 0.05. Complete results are in Table S2. NES follows the provider contrast convention and does not establish pathway activation.

Figure S2 (6/6) Complete significant KEGG GSEA overview

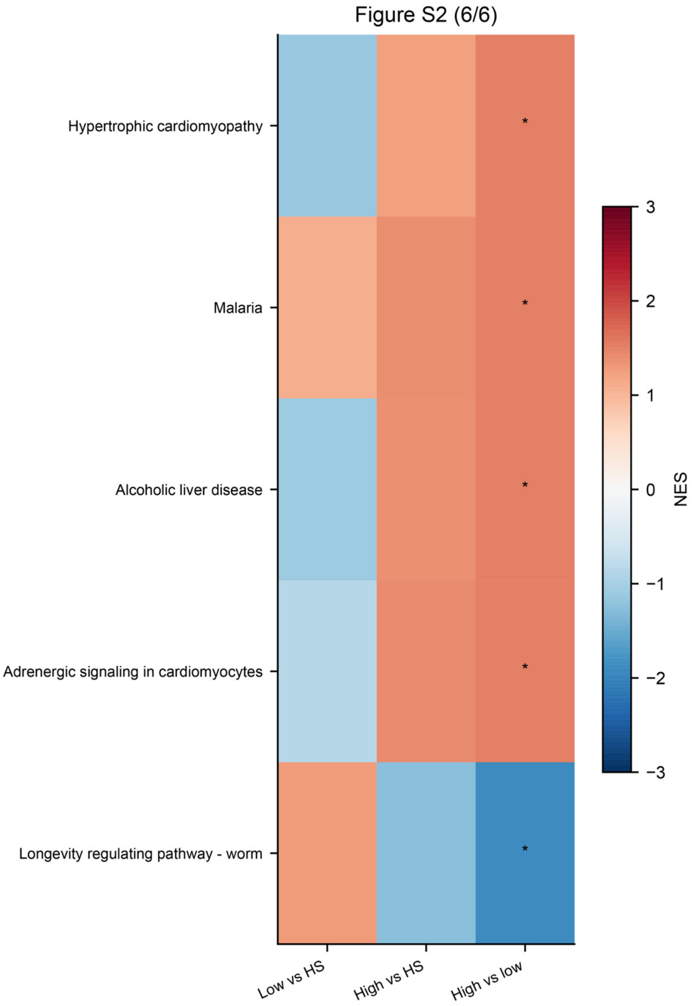

Figure S2 (6/6). Part 6 of the union of 125 pathways with provider-reported q < 0.05 in at least one contrast. Color represents NES; * indicates provider-reported q < 0.05. Complete results are in Table S2. NES follows the provider contrast convention and does not establish pathway activation.

Figure S3A Provider steroid-biosynthesis enrichment curve

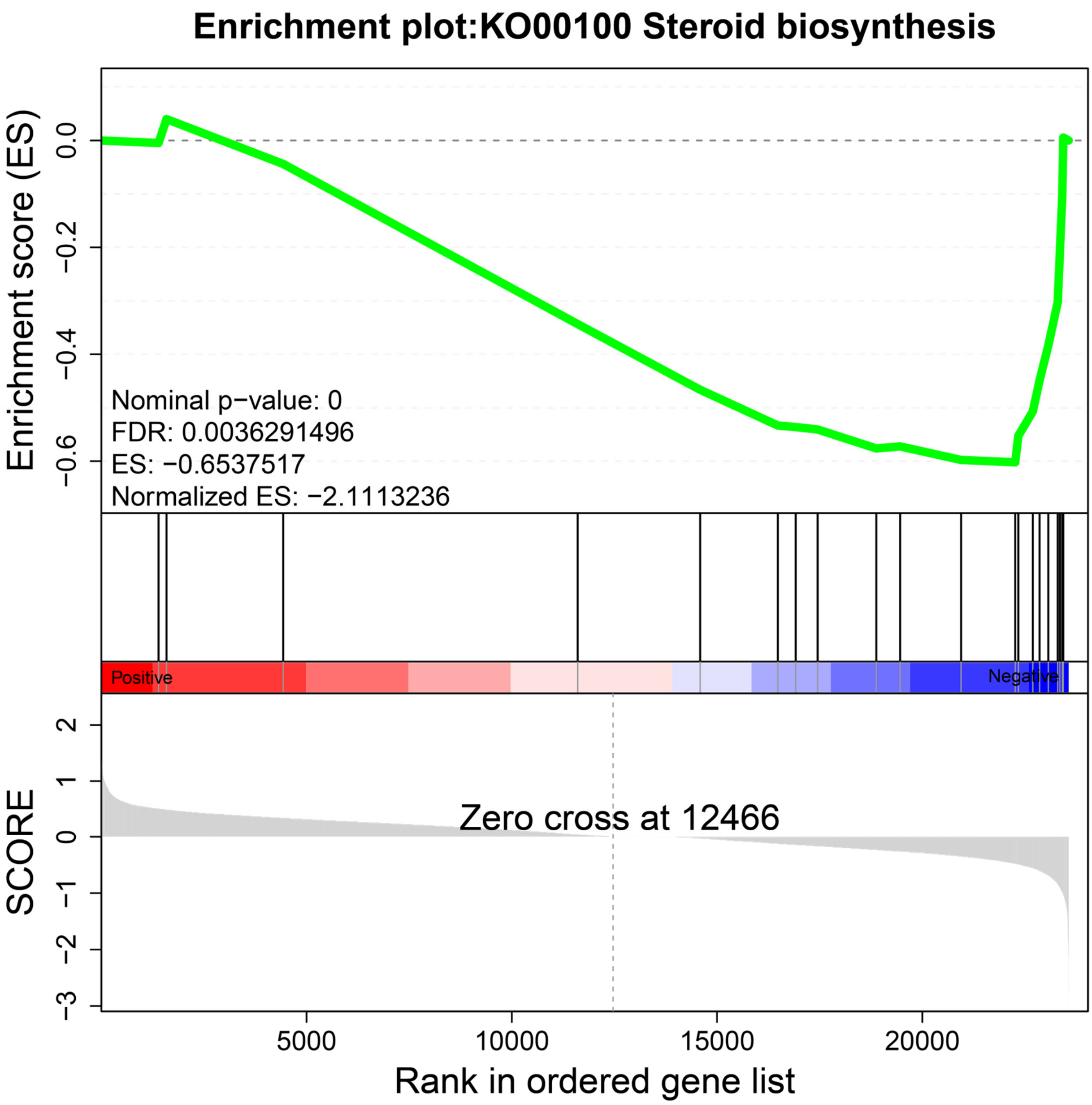

Figure S3A. High-dose versus HS comparison. Original curve retained; provider ES, NES and FDR are displayed.

Figure S3B Provider steroid-biosynthesis enrichment curve

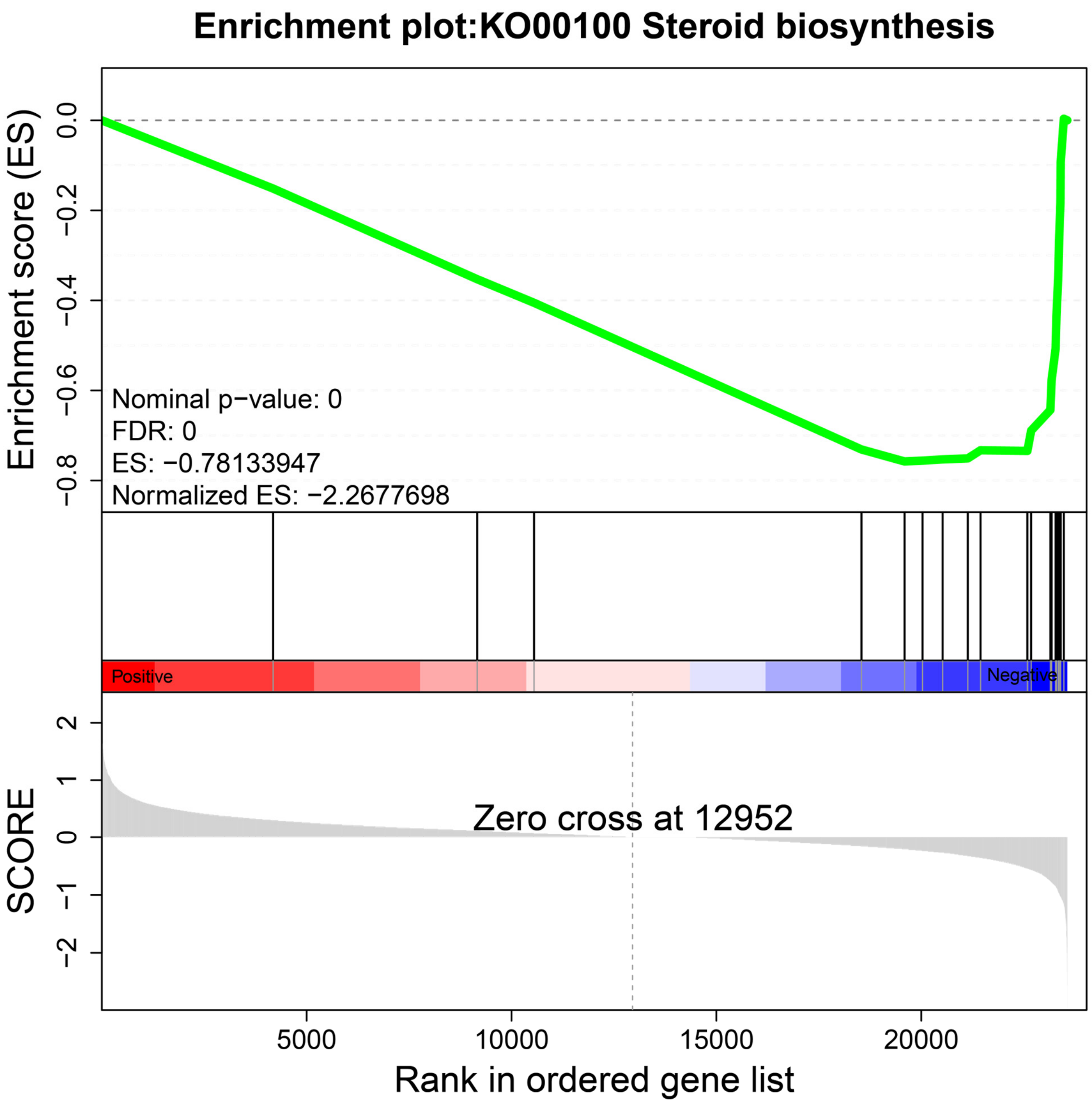

Figure S3B. Low-dose versus HS comparison. Original curve retained; displayed zero P or q values reflect provider output.

Figure S4 Rumen diversity, community composition and dispersion

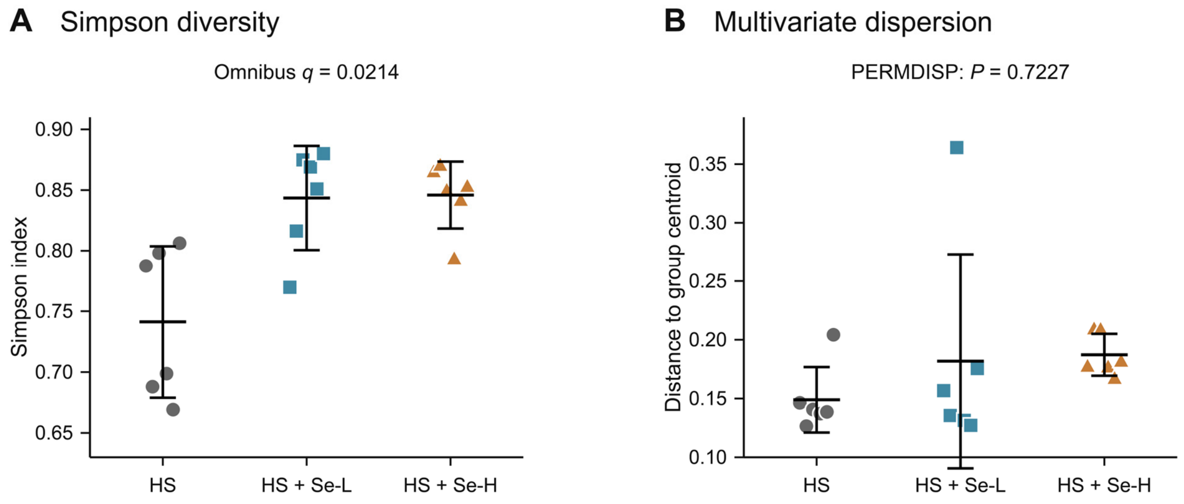

Figure S4. Additional rumen microbial diversity and dispersion analyses. (A) Simpson diversity differed among treatment groups after Benjamini–Hochberg correction across the three prespecified alpha-diversity measures (q = 0.0214). (B) Distances of individual samples to their treatment-group centroids based on genus-level Bray–Curtis dissimilarities. Multivariate dispersion did not differ significantly among groups (PERMDISP, P = 0.7227; 9,999 permutations). Points represent individual bulls, and black bars indicate means ± SD (n = 6 per treatment). HS, heat-exposed control; HS+Se-L and HS+Se-H, low- and high-dose selenium-enriched malt groups, respectively.

Figure S5 Selected exploratory microbiome–testis scatterplots

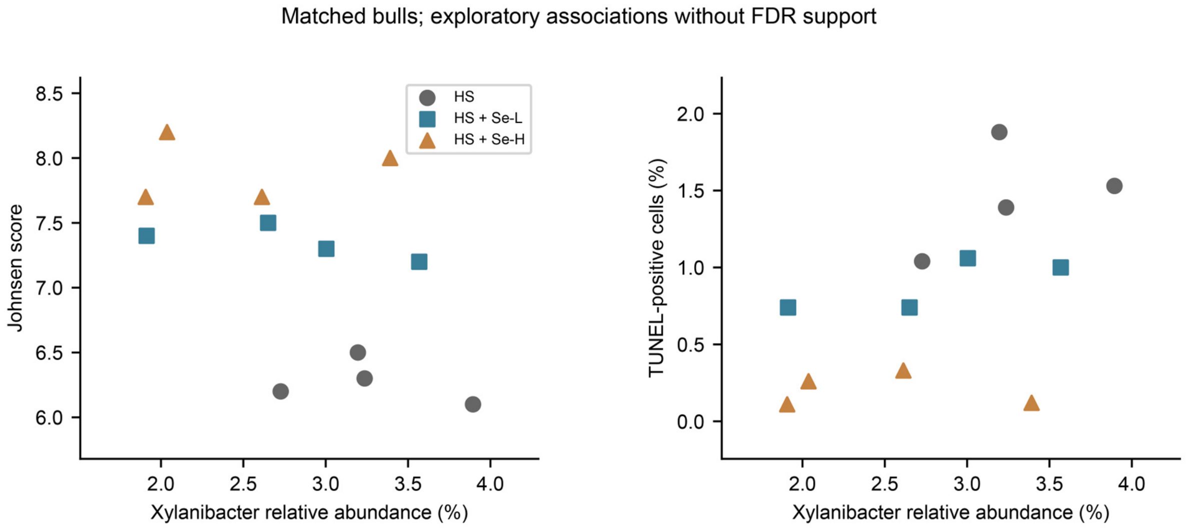

Figure S5. Xylanibacter abundance versus modified Johnsen score and TUNEL positivity in 12 matched bulls. These descriptive plots are not independent confirmatory tests. No association survived correction across the complete 160-test family; see Table S4.

Figure S6. Uncropped Western blot membrane images

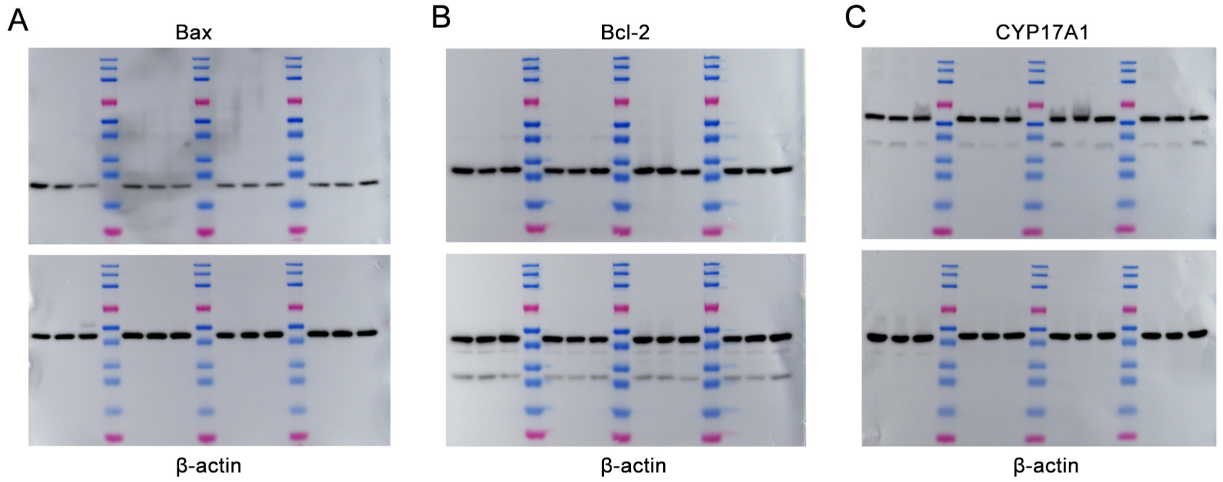

Figure S6. Uncropped Western blot membrane images supporting Figure 5. Full membrane images are shown with molecular-weight markers retained. Panels show (A) Bax (Servicebio, GB15007-100; 1:1,000) and its corresponding β-actin loading-control membrane (Servicebio, GB12001; 1:2,000), (B) Bcl-2 (Servicebio, GB154830-100; 1:1,000) and its corresponding β-actin membrane, and (C) CYP17A1 (Servicebio, GB112095-100; 1:1,000) and its corresponding β-actin membrane. Sample lanes are arranged from left to right as A1, B1, C1, marker, A2, B2, C2, marker, A3, B3, C3, marker, A4, B4, and C4, where A, B, and C denote the HS, HS+Se-L, and HS+Se-H groups, respectively.
